# Poly-lactic acid nanoparticles (PLA-NP) promote physiological modifications in lung epithelial cells and are internalized by clathrin-coated pits and lipid rafts

**DOI:** 10.1186/s12951-016-0238-1

**Published:** 2017-01-31

**Authors:** Camila Macedo da Luz, Matthew Samuel Powys Boyles, Priscila Falagan-Lotsch, Mariana Rodrigues Pereira, Henrique Rudolf Tutumi, Eidy de Oliveira Santos, Nathalia Balthazar Martins, Martin Himly, Aniela Sommer, Ilse Foissner, Albert Duschl, José Mauro Granjeiro, Paulo Emílio Corrêa Leite

**Affiliations:** 1 Laboratory of Bioengineering and in Vitro Toxicology, Directory of Metrology Applied to Life Sciences (Dimav), National Institute of Metrology Quality and Technology (INMETRO), Duque De Caxias, RJ Brazil; 2 Department of Molecular Biology, University of Salzburg, Salzburg, Austria; 3 Heriot-Watt University, Edinburg, UK; 4 Laboratory of Chemical Signaling in Nervous System, Biology Institute, Fluminense Federal University, Niteroi, RJ Brazil; 5 Laboratory of Biochemistry, State University Center of West Zone (UEZO), Rio de Janeiro, RJ Brazil; 6 Department of Cell Biology, University of Salzburg, Salzburg, Austria; 7 Dental School, Fluminense Federal University, Niteroi, RJ Brazil; 8 Av. Nossa Senhora das Gracas 50, LABET - Dimav, Predio 27, Duque de Caxias, Xerem, Rio de Janeiro, 25250-020 Brazil

**Keywords:** Nanoparticles, Drug delivery, Endocytosis, Lipid rafts, Clathrin-coated pits

## Abstract

**Background:**

Poly-lactic acid nanoparticles (PLA-NP) are a type of polymeric NP, frequently used as nanomedicines, which have advantages over metallic NP such as the ability to maintain therapeutic drug levels for sustained periods of time. Despite PLA-NP being considered biocompatible, data concerning alterations in cellular physiology are scarce.

**Methods:**

We conducted an extensive evaluation of PLA-NP biocompatibility in human lung epithelial A549 cells using high throughput screening and more complex methodologies. These included measurements of cytotoxicity, cell viability, immunomodulatory potential, and effects upon the cells’ proteome. We used non- and green-fluorescent PLA-NP with 63 and 66 nm diameters, respectively. Cells were exposed with concentrations of 2, 20, 100 and 200 µg/mL, for 24, 48 and 72 h, in most experiments. Moreover, possible endocytic mechanisms of internalization of PLA-NP were investigated, such as those involving caveolae, lipid rafts, macropinocytosis and clathrin-coated pits.

**Results:**

Cell viability and proliferation were not altered in response to PLA-NP. Multiplex analysis of secreted mediators revealed a low-level reduction of IL-12p70 and vascular epidermal growth factor (VEGF) in response to PLA-NP, while all other mediators assessed were unaffected. However, changes to the cells’ proteome were observed in response to PLA-NP, and, additionally, the cellular stress marker miR155 was found to reduce. In dual exposures of staurosporine (STS) with PLA-NP, PLA-NP enhanced susceptibility to STS-induced cell death. Finally, PLA-NP were rapidly internalized in association with clathrin-coated pits, and, to a lesser extent, with lipid rafts.

**Conclusions:**

These data demonstrate that PLA-NP are internalized and, in general, tolerated by A549 cells, with no cytotoxicity and no secretion of pro-inflammatory mediators. However, PLA-NP exposure may induce modification of biological functions of A549 cells, which should be considered when designing drug delivery systems. Moreover, the pathways of PLA-NP internalization we detected could contribute to the improvement of selective uptake strategies.

**Electronic supplementary material:**

The online version of this article (doi:10.1186/s12951-016-0238-1) contains supplementary material, which is available to authorized users.

## Background

The development and improvement of nanostructured materials with biocompatible characteristics are important objectives in the field of nanomedicine. Various NP have been developed for drug delivery, with gold nanoparticles (Au-NP) being the most used [18]. The advantages of Au-NP include the possibility of surface functionalization with a wide range of molecules and low or no cytotoxicity [54]. However, recent studies have performed extensive evaluation of Au-NP in several cell lines and have demonstrated their potential to induce cytotoxicity [9, 14, 15], endoplasmic reticulum stress, cleavage of cytoskeletal proteins [50], and susceptibility to cell death by apoptosis [37]. Au-NP demonstrated a slow clearance in mouse organs including muscle, liver, spleen, and kidney even 3 months post intravenous administration [77], and similar observations were made for rats [22]. This accumulation and slow clearance is a concern, as cells of the immune system can migrate into organs of the reticuloendothelial system, as well as resident immune cells, typically macrophages, may internalize Au-NP and release pro-inflammatory mediators, affecting the body homeostasis [56, 77]. Differences in the toxicity of Au-NP observed in existing studies could be in part a consequence of different physicochemical properties or due to different experimental designs when assessing their toxicity (e.g. differences in cell type, concentration, time point, assay sensitivity, NP shape and size, etc.) [51, 52, 60]. Thus, efforts are made to develop not only biocompatible, but also biodegradable NP.

Biodegradable and biocompatible nanocarriers for drug delivery have been prepared from different polymers and protocols in order to reduce cytotoxicity [43], which, in part, may be attributed to metal components of existing therapies or NP accumulation in organs and poor clearance [38]. Poly-lactic acid NP (PLA-NP) are a type of polymeric NP with potential applications in nanomedicine as carriers of drugs, proteins and genes [29, 75]. PLA-NP offers several benefits, such as sustainable therapeutic drug release over prolonged periods, due to their polymer matrix allowing the control of drug release kinetics [42]. In fact, the Food and Drug Administration (FDA) have approved the use of biodegradable materials, including PLA-NP, in humans [73]. Previous studies have evaluated the cytotoxic potential of PLA-NP in different cell types, such as CHO-K1 [46], HEK293 [6], retinal pigment epithelium [7], and MCF-7 and SK-BR-3 breast cancer cell lineages [43]. These studies concluded that PLA-NP were non-cytotoxic; these conclusions were based on the assessment of cell viability through measurements of mitochondrial activity, while other cellular stress parameters were not considered. Thus, the objective of the present study was to perform an extensive and thorough evaluation of PLA-NP effects, including cellular viability, analysis of intracellular ATP content, proliferation determined by electrical impedance, cytokine release, mRNA, and miRNA levels related to cell toxicity, stress and inflammation. In addition, we performed a proteomics approach aiming at investigating the composition of intracellular proteins that could be altered, inferring changes in biological function. We used the human A549 lung epithelial cell line, since the respiratory tract is one of the most important routes of administration and fast absorption of nanomedicine delivery systems. The inhalation route displays advantages such as avoidance of first-pass metabolism, fewer systemic side effects and circumvents the necessity for using needles. Previously, it has been shown that the cytotoxicity of polymeric NP loaded with cancer chemotherapeutics against A549 cells was greater compared to free drug, reinforcing the usefulness of polymeric NP as potential nanocarriers in lung cancer therapy. This was further reiterated when polymeric NP were administered to rats via inhalation, and higher drug concentrations were observed in the lung compared to plasma [2, 69].

In addition, this study investigated endocytic mechanisms related to PLA-NP uptake. Some well-characterized mechanisms of uptake pathways include endocytosis mediated by lipid rafts, caveolae, macropinocytosis and clathrin-coated pits. Lipid rafts are microdomains enriched with cholesterol and sphingolipids, and are involved in many functions such as compartmentalization of proteins related to intracellular signaling pathways, cellular communication, and endocytosis through still unknown mechanisms. Caveolae are structures similar to lipid rafts but formed by cell membrane invaginations that require caveolin proteins for their formation [17]. Macropinocytosis is an endocytic pathway for large contents of extracellular fluid that is dependent on cell membrane ruffling, and formation of macropinosomes. Clathrin-mediated endocytosis initiates from a cellular signal that promotes membrane invagination via clathrin-coated pit formation and vesicle release to the cytosol in a dynamin-dependent manner. Once in the cytosol, the clathrin coat is lost and uncoated vesicles fuse, resulting in early endosomes [20]. The interaction between NP and cell membranes is dependent on NP physicochemical characteristics and cell membrane properties, which in turn influences their intracellular trafficking and accumulation into organelles [1]. Recently, a study demonstrated that CCR5-targeted caveolin-1-functionalized NP were more efficiently internalized by non-phagocytic CD4+ T cells, when compared to untargeted NP, suggesting a potential strategy for drug delivery [25]. Therefore, the understanding of how to drive PLA-NP to target cell and the uptake mechanisms involved in its internalization through eukaryotic cell membranes are critical to design and develop selective strategies for reducing side effects and/or promoting an increase in active drug levels.

## Methods

### Nanoparticles and chemicals

Spherical monodispersed non- and green-fluorescent, Coumarin-6, PLA-NP (1.05 × 10^13^ particles/mL) were used [36]. Non-fluorescent NP were used in assays with light detection-dependent endpoints. Both NP were acquired from Institute of Biology and Chemistry of Proteins (IBCP), Lyon, France. RPMI 1640 medium supplemented with l-glutamine and fetal bovine serum (FBS) were purchased from Gibco BRL (Grand Island, NY, USA). Genistein, methyl-β-cyclodextrin (MCD) and amiloride hydrochloride hydrate were acquired from Sigma Chem. Co. (Saint Louis, MO, USA). Pitstop 2 was purchased from Abcam Biochemicals (Cambridge, UK).

### Nanoparticle characterization

The hydrodynamic diameter of green fluorescent (Coumarin-6, ex/em:444/505 nm) or non-fluorescent PLA-NP was determined by dynamic light scattering (DLS), after dilution in water. PLA-NP were also assessed in cell culture medium, at the same concentrations that were used in cell experiments. RPMI medium without FBS was used to dilute PLA-NP for TEM analysis. Particle suspensions were applied to TEM grids and left to dry prior to imaging using a LEO 912 AB Omega transmission electron microscope (Zeiss, Oberkochen) operated at 120 kV with a LaB6 cathode.

For analysis of NP hydrodynamic diameter and polydispersity index, PLA-NP were diluted in complete medium and incubated in cell culture flasks without cells following the same criteria as cell experiments. Then, 1 mL of sample was transferred to an appropriate cuvette for subsequent analysis by DLS and zeta potential in the Malvern Zetasizer Nano ZS apparatus (Malvern Instruments Ltd, Worcestershire, UK).

### Cell culture

A549 epithelial cells were mycoplasma-free (MycoAlert Mycoplasma Detection Kit-Lonza, Bazel, Switzerland), used for no more than 20 passages, and were seeded for experiments at 1.5 × 10^4^ cells/cm^2^. Cells were maintained in complete RPMI medium (10% FBS) according to ATCC Laboratories. PLA-NP were previously diluted at room temperature (RT), vortexed for 1 min and added to the cells at different concentrations (2, 20, 100 and 200 µg/mL, or 3.36 × 10^9^, 3.36 × 10^10^, 1.68 × 10^11^ and 3.36 × 10^1^ particles/mL, respectively) 24 h after cell seeding. After 24, 48 and/or 72 h of treatment, supernatants were collected and cells were used in experimental procedures. For the endocytosis inhibitors used in the uptake study, concentration- and time-dependent toxicity assessment was performed for each inhibitor and sublethal conditions were used in final experiments. It is important to highlight that each cell type has a different tolerance to chemicals and therefore subtoxic concentrations should always be determined in a case-by-case fashion. For the A549 cells used here, the subtoxic concentrations used were 200 µM genistein, 2 mM MCD, 1.5 mM amiloride, and 12.5 µM pitstop 2 [4, 8, 28, 35]. All inhibitors were applied to cells for 40 min, except for pitstop 2, which was applied for only 10 min. After incubation with endocytosis inhibitors, the cells were washed and further incubated with 20 µg/mL PLA-NP for 1 or 4 h. In the cytotoxicity and viability assays, the positive control for cell death was performed by incubation with 1% Triton X-100 (TX-100) for 20 min. Absorbance, fluorescence and luminescence were measured in a Tecan Infinite 200PRO microplate reader. Intrinsic NP interference was measured for each experiment, using the respective assay reagents and excluding cells; these values were subtracted from experimental groups.

### MTT assay

After PLA-NP exposure, cells were washed twice with PBS and 100 µL of complete medium containing 0.15 mg/mL MTT (3-(4,5-dimethyl-2-thiazolyl)-2,5-diphenyl-2H-tetrazolium bromide, Sigma Chem. Co., Saint Louis, MO, USA) was added. After 3 h, supernatants were discarded, cells washed twice with PBS, and samples homogenized with 100 µL DMSO. Absorbance was measured at 480 nm with reference readings at 690 nm.

### Intracellular ATP (adenosine triphosphate) analysis

Cells were lysed with CelLytic M lysis buffer containing 1% protease inhibitor cocktail, according to the manufacturer’s recommendations (Molecular Probes, Eugene, OR, USA). Samples were transferred to flat-black 96 multi-well plates followed by the addition of the standard reaction solution containing recombinant firefly luciferase and its substrate d-luciferin (ATP Determination Kit, Molecular Probes, Eugene, OR, USA). Plates were incubated for 10 min at 28 °C and luminescence was determined.

### Lactate dehydrogenase (LDH) release assay

LDH release was determined using the colorimetric CytoTox 96 Cytotoxicity Assay kit (Promega, Madison, WI, USA). A positive control was performed by treating cells with 1% TX-100 for 20 min. After PLA-NP treatments, 30 µL of cell supernatants were transferred to new 96-well plates followed by the addition of 30 µL of substrate solution. After 20 min of incubation in the dark at RT, 30 µL of stop solution was added to each sample. Color development was proportional to the number of cells with disruption of plasma membrane. Absorbance was measured at 490 nm.

### Real-time electrical impedance cell monitoring

Cells were seeded in 96-well E-Plate View xCELLigence RTCA SP system containing gold microelectrodes on well bottoms (ACEA Biosciences, San Diego, CA, USA). This label-free methodology is sensitive enough to measure slight differences of cell response under a wide range of stimuli. After cell seeding for 24 h, the instrument was programmed to monitor cell proliferation and cytotoxicity each hour during 96 h, through electrical impedance analysis. PLA-NP were added after the first 24 h growth and cells were monitored for a further 72 h. Cell impedance is represented by cell index (CI) = (Z_i_ − Z_0_) [Ohm]/15[Ohm], where Z_0_ is background resistance and Z_i_ is resistance at an individual time point. Normalized cell index was determined as cell index at a specific time point (CI_ti_) divided by cell index at normalization time point (CI_nml_time_).

### Proteomics assay

Cells were treated with 20 µg/mL PLA-NP for 24 h, protein was extracted using a lysis buffer (CelLytic M) with 1% protease inhibitor cocktail (Sigma, Saint Louis, MO, USA). Soluble proteins were recovered and quantified by 2-D Quant kit (GE Healthcare, USA) resulting in an average of 3 µg/µl. Then, 50 ug of each sample was processed for mass spectrometry analysis. Sequencing grade porcine trypsin (Promega, USA) was used to digest proteins that were previously reduced and alkylated by incubation with 10 mM dithiothreitol (DTT) and 55 mM iodoacetamide (IAA), respectively. Tryptic peptides were analyzed by liquid chromatography tandem mass spectrometry (LC–MS/MS) using a nanoACQUITY UPLC system coupled to a Synapt G1 High Definition Mass Spectrometer (Waters, USA). Nanoflow ESI source was applied with a lock spray source for lock mass measurements during all chromatographic runs. Digested proteins were desalted using a Trap Symmetry C18 column (Waters, USA). Mixture of trapped peptides was eluted with a water/ACN 0.1% formic acid gradient through a Symmetry C18 (150 µm) capillary column. Data were acquired in expression mass spectrometry mode (MSE). Samples were initially run once in sequence for normalization by ion counting method. Then, normalized samples were analyzed in triplicates by mass spectrometry. The LC–MS/MS data were processed by ProteinLynx 2.0 software (Waters, USA) to a search against a protein human database from UniProt Protein Knowledgebase (http://www.uniprot.org/uniprot/?query=Human&sort=score).

### Quantitative real-time polymerase chain reaction (qPCR)

Total RNA was extracted from A549 cells treated for 72 h with 20 µg/mL PLA-NPs using miRNeasy mini kit (Qiagen, USA) according to manufacturer’s instructions. The amount and purity of total RNA were evaluated with a UV spectrophotometer (NanoDrop 2000, Thermo Fisher Scientific Inc, MA, USA), by A260/280 and 260/230 ratios, considering the cut-off values equal or greater than 2.0 and 1.8, respectively. The integrity of the RNA extracted was evaluated in bleach gel stained with gel red (Biotium, CA, USA). The material was stored at −80 °C until ready for gene expression analysis. For TP53, PCNA and PPARγ mRNA expression analysis, all the reagents were purchased from Applied Biosystems, USA. The qPCR was performed using the AgPath-ID one-step RT-PCR kit. Briefly, purified RNA was reverse transcribed and amplified. Individual mRNAs were quantified with the 7500 Real-Time PCR System (Applied Biosystems, USA) using TaqMan Gene Expression Assays (TP53-Hs01034249_m1, PCNA- Hs00427214_g1, PPARγ-Hs01115513_m1). The reference genes CASC3 (cancer susceptibility candidate gene 3) and RPL10 (that encodes the 60S ribosomal protein L10) were used as internal controls. Thermal cycling comprised of a 10 min RT step at 45 °C, and a 10 min initial PCR activation step at 95 °C (AmpliTaq Gold activation), followed by 40 cycles of 95 °C for 15 s and 60 °C for 45 s. For micro-RNA (miRNA) analysis, the cDNA synthesis was performed using TaqMan MicroRNA Reverse Transcription Kit (Applied Biosystems, USA). Custom PCR Array plates containing specific primers were used to detect miRNAs that play a role in inflammation and cell death processes: let-7a, miR21, miR125b, miR155. miR17-5p was selected as internal control. The cycle parameters comprised of 10 min at 95 °C, followed by 40 cycles of 15 s at 95 °C and 1 min at 60 °C, according to the manufacturer. The 2^−ΔΔCt^ method was performed for comparing relative fold expression differences.

### Multiplex analysis of secreted products

Determination of proteins secreted by A549 cells upon exposure of PLA-NP was carried out, using Luminex xMAP magnetic technology, for the following analytes: IL-1β, IL-1ra, IL-2, IL-4, IL-5, IL-6, IL-7, IL-8, IL-9, IL-10, IL-12 (p70), IL-13, IL-15, IL-17, eotaxin, bFGF, GCSF, GM-CSF, IFN-γ, IP-10, MCP-1 (MCAF), MIP-1α, MIP-1β, PDGF-BB, RANTES, TNFα, VEGF. The positive control used was 20 ng/mL TNFα. The assays were performed following the manufacturer’s recommendations. In brief, after calibration and validation of Bio-Plex Magpix (Biorad Laboratories Inc., Hercules, CA, USA), reagent reconstitution and standard curve preparation, magnetic beads were added to each well of the assay plate. Each step was preceded by washing steps using an automated Bio-Plex Pro wash station (Biorad Laboratories Inc., Hercules, CA, USA). Beads were added followed by supernatant samples, standard and controls and incubated in the dark for 1 h at 350 rpm. Samples were incubated with detection antibodies, using the same incubation parameters. Streptavidin-PE was added and incubated in the dark for 30 min at 350 rpm. Finally, magnetic beads were resuspended in assay buffer, agitated at 1200 rpm for 30 s and read in the Bio-Plex Magpix apparatus. Assay interference was controlled by incubation of the PLA-NP with a standard series of IL-8 determined by ELISA and of IL-12 and VEGF determined by Magpix technology suggesting that PLA-NP do not bind the secreted products (data not shown).

### Caspases-3/7 assay

Cells were previously incubated with non-fluorescent PLA-NP in a black flat-bottom 96 multi-well plate for 72 h at 37 °C, followed by PBS washing and incubation with 100 nM staurosporine (STS) for 24 h at 37 °C. Positive controls were performed by incubating cells with STS only. The Apo-ONE Homogeneous Caspases-3/7 (Promega, Madison, WI, USA) assay was performed following manufacturer recommendations. Fluorescence intensity was quantified using 485/528 nm excitation/emission in a Tecan Infinite 200PRO microplate reader.

### Fluorescent confocal analysis

After exposure to PLA-NP, cells were washed with PBS and fixed with 4% formaldehyde for 15 min at room temperature. Cells were permeabilized and blocked with 1% bovine serum albumin for 1 h. F-actin was stained using rhodamine-labelled phalloidin (1:100 dilution of stock, 6.6 µM in 0.1% BSA) for 1 h at RT and slides mounted with Prolong Gold Antifade reagent with DAPI (4′,6-diamidino-2-phenylindole, both from Molecular Probes, Eugene, OR, USA). For lipid raft staining, the Alexa Fluor 594-coupled cholera toxin subunit B (CTX, 1 µg/mL, Molecular Probes) was used, a lipid raft marker which binds to ganglioside GM1. A549 cells were incubated for 10 min at 4 °C with CTX, followed by PLA-NP treatment for 60 min at 37 °C and fixed as previously described. Confocal imaging was performed with a Leica TCS SP5 confocal laser scanning microscope (Mannheim, Germany) coupled to a DMI 6000B inverted microscope and a 63× water immersion objective with an 1.2 numerical aperture. For the excitation of PLA-NP fluorescence, the 488 nm line of the Argon laser was used and the emitted light was detected at 500–537 nm range. DAPI was excited at 405 nm (diode laser) and detected between 430 and 472 nm. Rhodamine and Alexa Fluor 594 were excited at 561 nm (diode pumped solid-state laser) and the detection window was 589-679 nm. The adjustment of gain settings was performed on untreated control samples and Z-stack images were acquired with a step size between 130 and 210 nm.

### Flow cytometry

Endocytosis inhibitors were used and PLA-NP treatments were performed as previously described [4, 8, 28, 35]. After PLA-NP treatment, cells were washed with PBS and fresh medium was applied for 30 min in normal cell culture conditions. Then, cells were washed and trypsinized, suspended in cell culture medium containing FBS and centrifuged. Cells were suspended in PBS containing 2% FCS and analyzed by Canto II flow cytometer (Becton, Dickinson and Company, Franklin Lakes, NJ, USA). Green fluorescent cell populations in PLA-NP exposed cells, with no inhibitors, were used as positive controls, and MFI and percentage changes of these gated cells were used for analysis.

### Statistical analysis

Concentration of each secreted product was quantified with xPONENT software version 4.2 (Biorad Laboratories Inc., Hercules, CA, USA). Flow cytometry analysis was performed on BD FACSDiva v8.0. GraphPad Prism 5 (GraphPad software Inc., La Jolla, CA, USA) was used to calculate mean and standard errors of the others assays. Normality tests were performed and One-way ANOVA and unpaired *t* test were applied to obtain statistical significance of means. Differences were considered statistically significant at the 0.05 level of confidence.

## Results

### PLA-NP characteristics

**Fig. 1:**
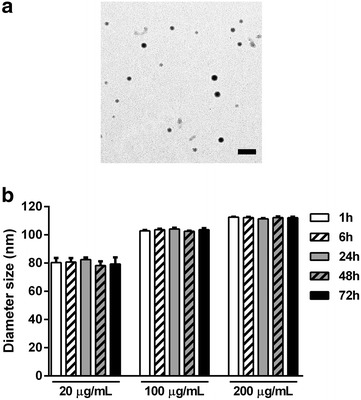
PLA-NP characteristics. **a** Representative images obtained by TEM of 2 μg/mL PLA-NP.* Scale bar* 200 nm. **b** Z-average hydrodynamic diameter values of PLA-NP at 20, 100 and 200 µg/mL diluted in complete medium, i.e. same conditions as in cell culture, and analyzed by DLS after 1, 6, 24, 48 and 72 h of incubation. Results are expressed as mean (±SD)

Micrographs of PLA-NP were acquired by TEM (Fig. 1a). Hydrodynamic diameters of PLA-NP in water, assessed by DLS, were 63 and 66 nm for non- and green-fluorescent PLA-NP, respectively, and zeta potential analysis indicated a −49 mV surface charge. Under cell culture conditions (without cells), PLA-NPs were shown the slightly increase in size compared to samples suspended in water, and moreover, a small increase in PLA-NP hydrodynamic diameter was observed to be both time- and concentration dependent. When incubated at 20 µg/mL, the z-average hydrodynamic diameter of PLA-NP was shown to be 78.2 ± 1.5 nm after 1 h incubation and 82.4 ± 3 nm after 72 h, whereas at 100 µg/mL the diameter increased to 102.6 ± 0.4 nm after 1 h and 104.1 ± 0.9 nm after 72 h, and further increased when PLA-NP were incubated at 200 µg/mL, to 111.4 ± 0.5 nm after 1 h and 112.6 ± 0.3 nm after 72 h (Fig. 1b). However, the polydispersity index did not show differences over time (1–72 h) indicating a stable particle suspension, but was found to reduce dependent upon NP concentration (20 µg/mL, 0.592 ± 0.03; 100 µg/mL, 0.265 ± 0.01; and 200 µg/mL, 0.196 ± 0.01), fluorescent and non-fluorescent PLA-NP were found to be comparable. This data suggest that PLA-NP were stable, regarding agglomeration, in cell culture medium up to 72 h.

### Effects of PLA-NP on cell viability, cytotoxicity and proliferation

**Fig. 2:**
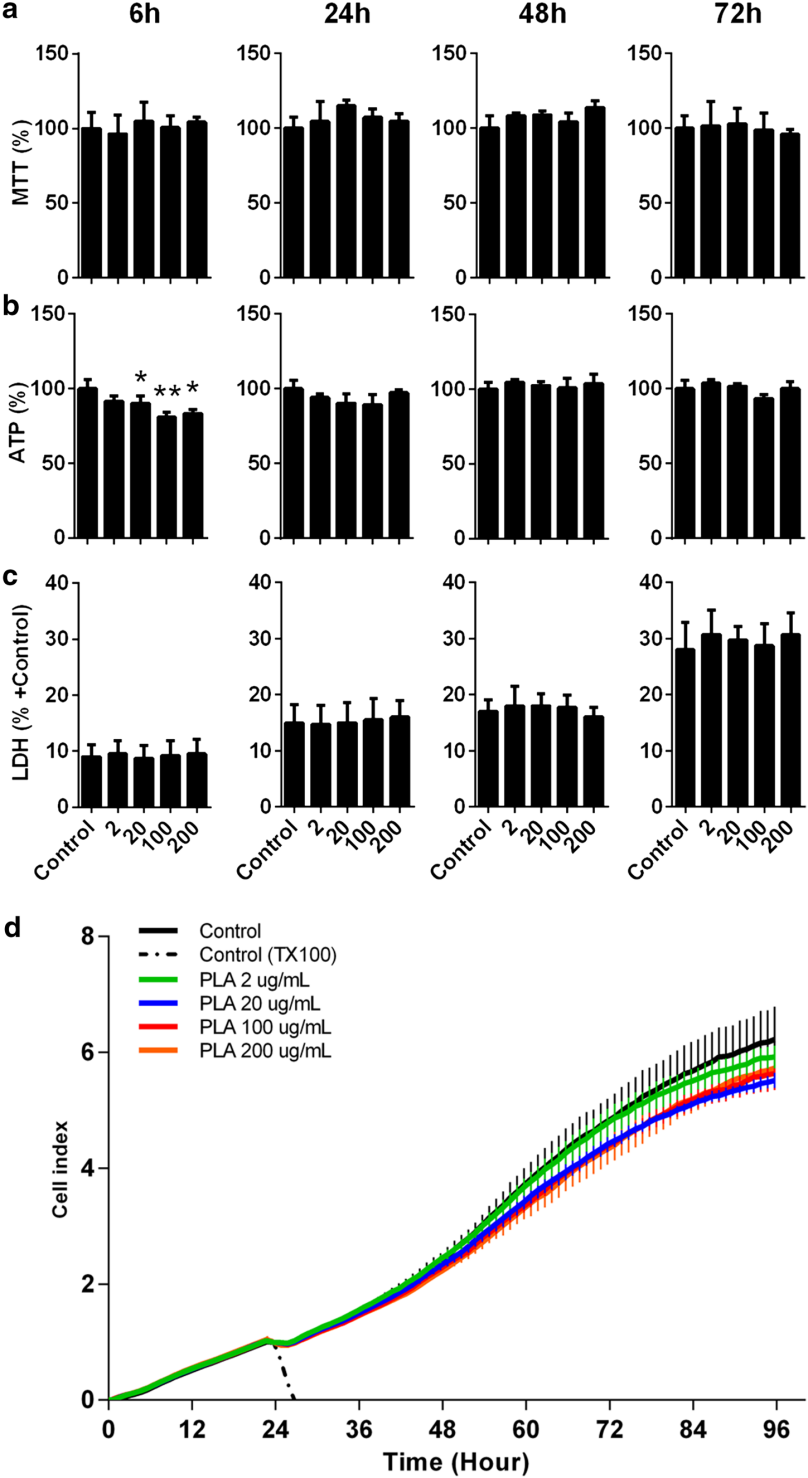
Effects of PLA-NP on cell viability, cytotoxicity and proliferation. *Graphs* showing the effects of PLA-NP at different concentrations (2, 20, 100 and 200 µg/mL) in A549 cells after 6, 24, 48 and 72 h upon **a** MTT conversion into formazan, **b** intracellular ATP levels, and **c** LDH release compared to ‘+ Control’. **d** Real-time electrical impedance cell monitoring of A549 cells treated with PLA-NP at different concentrations after 24 h of cell seeding and maintained to 96 h. ‘Control’ corresponds to A549 cells without treatment, and ‘+ Control’ means ‘positive control of total LDH release’ treated with 1% TX100 for 20 min. Results are expressed as mean (±SD). Each experimental group corresponds to three independent experiments performed in four replicates. One-way ANOVA test with Bonferroni’s multiple comparisons test compared to Control group (*p < 0.05, **p < 0.01)

Cell viability, proliferation rates and cytotoxicity were assessed after exposure to PLA-NP at different concentrations (2, 20, 100 and 200 µg/mL) for different times (6, 24, 48 and 72 h). Determination of MTT conversion into formazan did not show loss of viability in any exposure condition (Fig. 2a). A slight reduction of intracellular ATP level was observed only after 6 h exposure at 20 µg/mL (9.8 ± 3.9%, *p* < 0.05), 100 µg/mL (19.1 ± 3.4%, *p* < 0.01) and 200 µg/mL (16.5 ± 3.2%, *p* < 0.05) (Fig. 2b). In addition, LDH release did not increase in response to any NP exposure condition compared to control cells (Fig. 2c). Analysis of cell proliferation was determined by real-time electrical impedance monitoring. PLA-NP did not induce alterations in cell proliferation or adhesion at the concentrations studied (Fig. 2d). Altogether, these data suggest that PLA-NP caused no cytotoxicity, and did not induce loss of viability or affect cell proliferation.

### Impact of PLA-NP on cytokine secretion

**Fig. 3:**
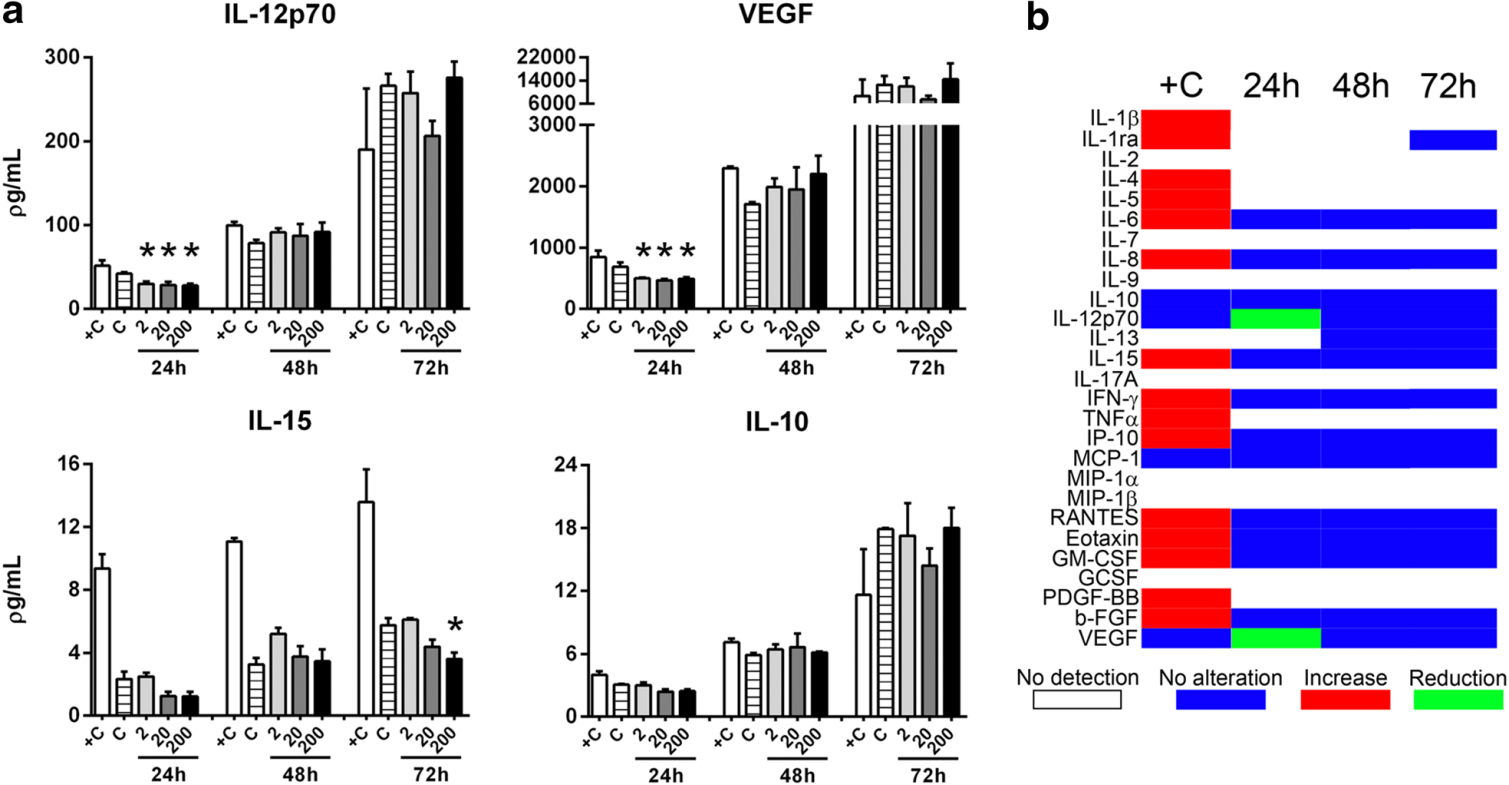
Impact of PLA-NP on cytokine secretion. **a** *Graphs* showing the effects of PLA-NP at different concentrations (2, 20 and 200 µg/mL) in A549 cells after 24, 48 and 72 h upon IL-12p70, VEGF, IL-15 and IL-10 levels. **b** *Heat graph* showing all secreted products analyzed after 20 µg/mL PLA-NP treatment during 24, 48 and 72 h. ‘+C’ means positive control corresponding to A549 cells treated with 20 ng/mL TNFα for 24 h. Each experimental group corresponds to the analysis of three independent experiments. One-way ANOVA test with Bonferroni’s multiple comparisons test compared to respective Control group (*p < 0.05)

Multiplex analysis of secreted products from A549 cells after PLA-NP exposure during 24 h did not show increased levels of any soluble mediator, but decreased levels of the following mediators were observed: IL-12p70 at 2, 20 and 200 µg/mL, reducing 28.5 ± 8.2%, 31.9 ± 9.7% and 33.6 ± 7.3%, respectively, compared to control (*p* < 0.05 for all comparisons). VEGF levels also decreased after 24 h, reaching 27.6 ± 10.3%, 32.3 ± 10.6% and 28.7 ± 10.8%, for treatments of 2, 20 and 200 µg/mL, respectively (*p* < 0.05 for all comparisons). IL-15 cytokine levels were reduced only after 72 h at 200 µg/mL PLA-NP exposure (37.6 ± 10.3%, *p* < 0.05). PLA-NP treatment did not change the levels of the anti-inflammatory IL-10 in any condition. The other mediators were unchanged or not detected in all conditions (Fig. 3a). The pattern of all secreted products after 24, 48 and 72 h of treatment with 20 µg/mL PLA-NP is shown in the heat map. The positive control group induced by TNFα showed increased levels of most analyzed mediators (Fig. 3b).

### Impact of PLA-NP on intracellular protein, mRNA, and miRNA levels related to cell toxicity, stress and inflammation, and apoptosis susceptibility

**Fig. 4:**
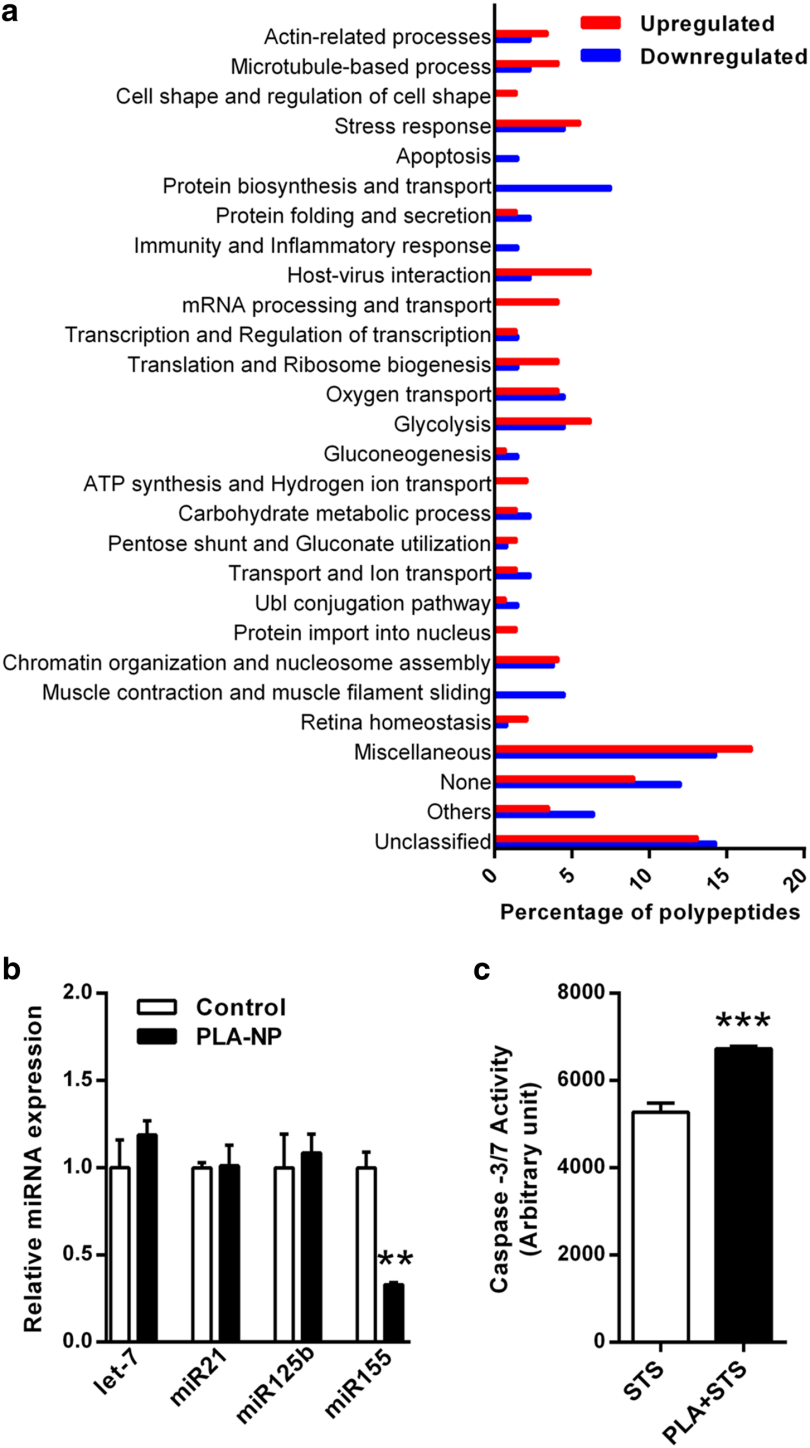
Impact of PLA-NP on intracellular proteins levels and miRNA related to cell toxicity, stress and inflammation. **a** Percentage of polypeptides from proteins into biological functions regulated in response to 20 µg/mL PLA-NP treatment for 24 h. GO (Gene Ontology) functions are ‘Miscellaneous’ includes proteins with more than 2 biological functions. ‘None’ means none information found. ‘Unclassified’ comprises proteins without described biological process but only molecular function. Functions with under 1% percentage were grouped in ‘Others’ category which includes: autophagy; bicellular tight junction; blood coagulation and hemostasis; camera-type eye development/in utero embryonic development; cell cycle and mitosis; cell redox homeostasis; differentiation; neurogenesis; ectoderm development; mineral balance; N-glycan processing; nucleotide metabolism; pyridoxal 5′-phosphate salvage. **b** miRNA analysis of A549 cells exposed to 20 µg/mL PLA-NP after 72 h incubation. Each experimental group corresponds to three independent experiments. Student *t*-test compared to Control group (**p < 0.01). **c** Apoptosis susceptibility of A549 cells exposed to 20 µg/mL PLA-NP during 72 h followed by 24 h treatment with 100 nM STS. Each experimental group corresponds to the analysis of three independent experiments performed in duplicates. Student *t* test compared to STS group (***p < 0.001). Results are expressed as mean (±SEM)

Proteomic evaluation showed 278 polypeptides were either up- or down-regulated in response to 20 µg/mL PLA-NP. Amongst these, 145 were completely or partially upregulated, including sequences for heat shock proteins (HSPs), histones, hemoglobins, heterogeneous nuclear ribonucleoproteins and others (Additional file 1: Table S1). However, 133 polypeptides were completely or partially downregulated, including actins and actinin subunits, elongation factors, tropomyosin polypeptides and others (Additional file 2: Table S2). Biological functions of all regulated sequences were determined according to GO (Gene Ontology) annotation. Upregulated dominant functions comprised of glycolysis, stress response and host-virus interaction, whereas downregulated functions were related to protein biosynthesis and ‘other’ functions. In both differentially regulated groups, proteins were detected that have variable or non-described function (described as ‘Miscellaneous’ and ‘None’ categories), as well as ‘Unclassified’ in terms of their related biological processes (Fig. 4a). Major upregulated ‘Unclassified’ proteins were related to nucleic acid, calcium and ATP binding and structural molecular activity (Additional file 1: Table S1). Downregulated ‘Unclassified’ proteins comprised of those related to oxidoreductase activity and calcium and ATP binding (Additional file 2: Table S2).

The effects of 20 µg/mL PLA-NP on mRNA expression was evaluated after 72 h cell-exposure. The mRNA levels of TP53 (tumor suppressor), PCNA (associated with cell proliferation) as well as PPARg (involved in inflammation pathway) were not altered by PLA-NP (data not shown). Moreover, amongst the miRNAs associated with cell death and inflammation processes analyzed, only miR155 was downregulated after PLA-NP exposure (67.1 ± 9.1%, *p* < 0.01) (Fig. 4b).

Since miR155 expression levels were reduced upon PLA-NP treatment, and this miRNA is involved with cellular resistance in lung epithelial A549 cells [78], it was considered that PLA-NP exposure could potentiate Caspases-3/7 activation in response to a agent known to induce apoptotic cell death, such as STS. It was shown that by pretreating A549 cells with 20 µg/mL PLA-NP for 72 h, apoptosis in response to 100 nM STS was increased, with caspases-3/7 activation increasing by 27.6 ± 4.0% (*p* < 0.001) compared to control cells (STS-stimulation only) (Fig. 4c).

### Mechanisms involved in PLA-NP uptake

**Fig. 5:**
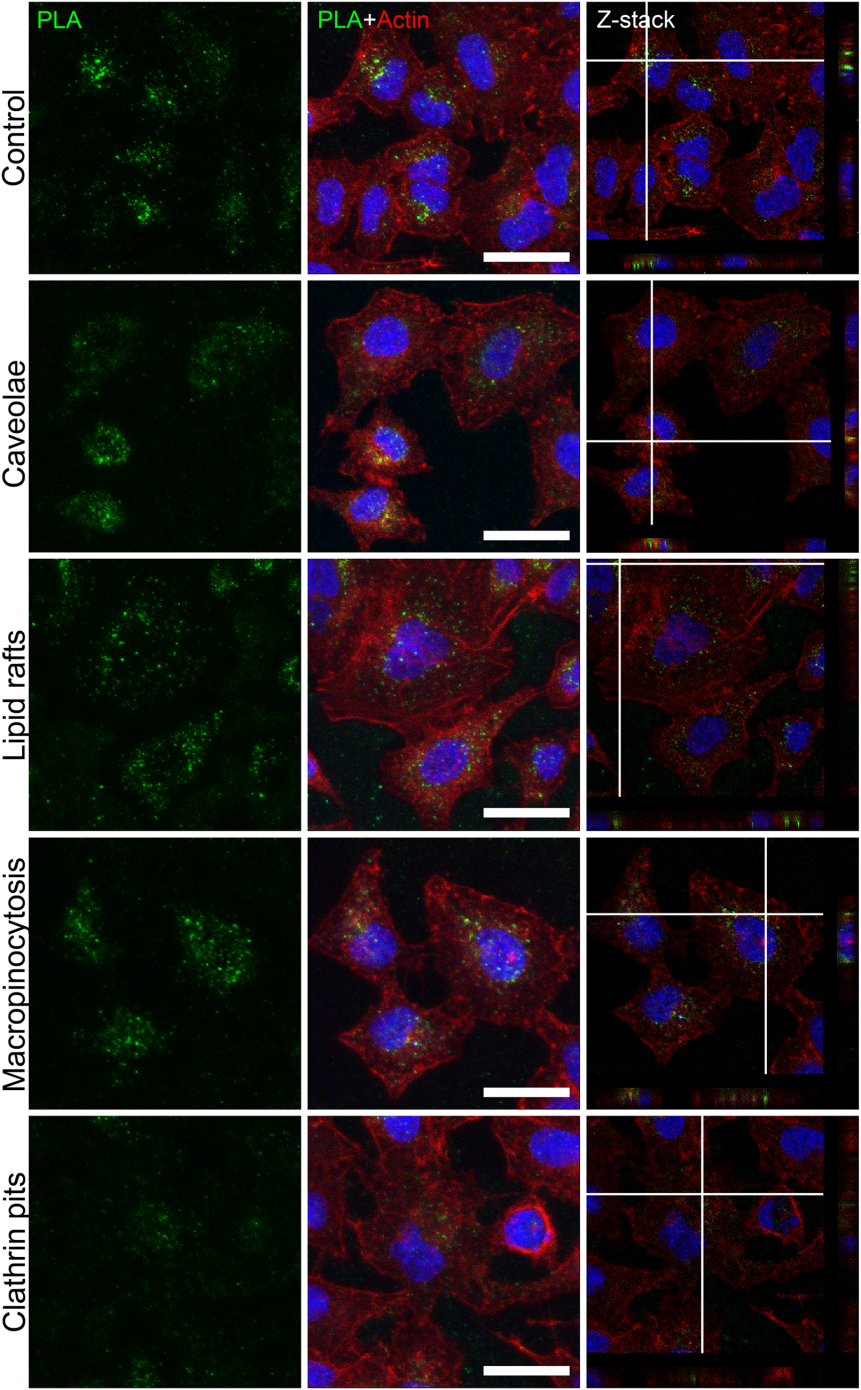
Endocytic pathways involved in PLA-NP uptake. Representative images acquired as Z-stacks by confocal microscopy of three independent experiments. A549 cells were treated with inhibitors for different endocytic pathways followed by 60 min incubation with 20 µg/mL PLA-NP. On the *left*, the inhibited pathways are indicated, instead of the inhibitors used. *Green dots* refer to PLA-NP, *red* and *blue* staining to actin and nuclei, respectively. The *mid-lane* represents maximum projections of at least 25 optical sections of the Z-stacks. The images on the *right lane* are single-slices from the corresponding Z-stacks and the cross-hair set indicates evidence of the PLA-NP internalization (the cross-section of the sample in the yz-plane is shown on the *right* and in the xz-plane at the *bottom*). *Scale bar* 30 µm

**Fig. 6:**
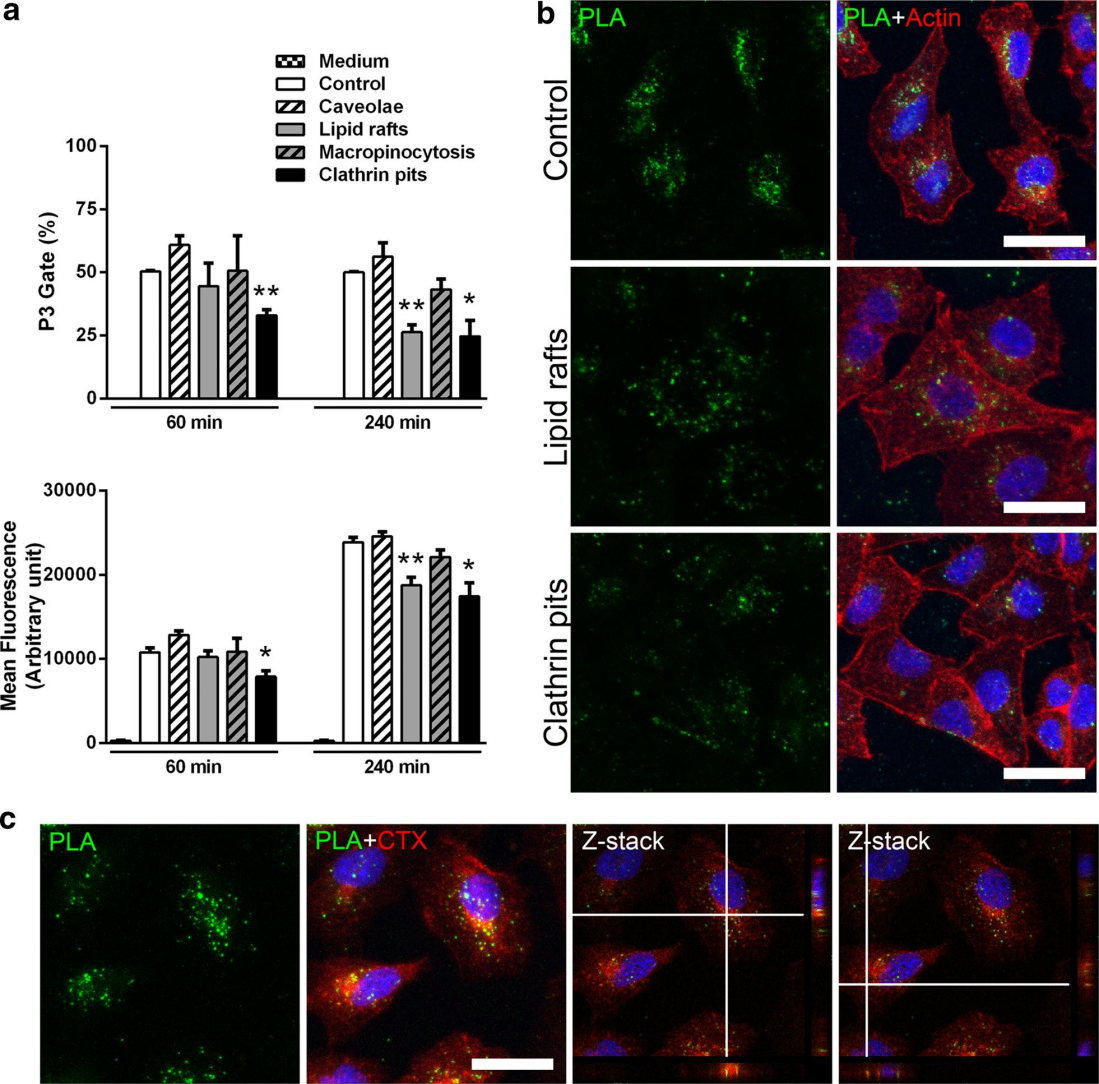
Flow cytometry and confocal analysis of PLA-NP uptake. **a** Flow cytometry analysis of A549 cells treated with the endocytic pathways inhibitors followed by 60 or 240 min incubation with 20 µg/mL PLA-NP. ‘P3 Gate’ refers to A549 half population in green fluorescent gate region. ‘Mean Fluorescence’ reflects the analysis of ‘P3 Gate’. Each experimental group corresponds to three independent experiments performed in two replicates. Student *t* test compared the experimental condition to respective Control groups (*p < 0.05, **p < 0.01). Results are expressed as mean (±SEM). **b** Representative images of three independent experiments acquired by confocal microscopy of A549 cells treated with lipid rafts and clathrin pits inhibitors followed by 240 min incubation with 20 µg/mL PLA-NP. *Green dots* refers to PLA-NP, *red* and *blue* staining to actin and nuclei, respectively. **c** Representative images of three independent experiments showing the co-localization of the lipid raft marker CTX-Alexa Fluor 594 and PLA-NP in A549 cells. *Green dots* refer to PLA-NP, *red* and *blue* staining to ganglioside GM1-bound CTX and nuclei, respectively. Cross-hair lines in the Z-stack images evidence *green* and *red* fluorescence overlapping, hence co-localization. *Scale bar* 30 µm

Aiming to investigate the mechanisms involved in PLA-NP uptake, different endocytic pathways were inhibited with subtoxic yet according to previous reports still functional concentrations of chemicals such as genistein (caveolae), MCD (methyl-β-cyclodextrin) (lipid rafts), amiloride (macropinocytosis) and pitstop 2 (clathrin-coated pits) [4, 8, 28, 35]. Actin staining (red) demonstrated that treatments did not affect cell shape and adhesion. In order to distinguish between internalized PLA-NP and PLA-NPs situated outside the cells, we estimated their position relative to nuclei and actin strands by performing confocal Z-stack imaging. Following 60 min incubation with 20 µg/mL PLA-NP (green dots) it was evident that PLA-NP were internalized in all conditions. However, a reduction of their internalization after inhibiting clathrin-mediated endocytosis was observed (Fig. 5). These conditions were also assessed by flow cytometry, where clathrin-mediated endocytosis inhibition showed reduction in PLA-NP uptake (34.5 ± 4.5%, *p* < 0.01) and mean fluorescence (26.7 ± 7.8%, *p* < 0.05) after 60 min of PLA-NP treatment. When PLA-NP incubation was performed for 240 min, both inhibition of clathrin-mediated endocytosis and lipid rafts disruption reduced PLA-NP internalization. For clathrin-mediated endocytosis, a reduction of 50.7 ± 12.6% (*p* < 0.05) (mean fluorescence reduction of 27 ± 7.1%, *p* < 0.05) was observed upon inhibition of this mechanism, and for lipid rafts inhibition, a 47.3 ± 4.8% (*p* < 0.01) (mean fluorescence reduction of 21.3 ± 4.4%, *p* < 0.01) reduction in uptake was observed. Inhibition of caveolae-mediated endocytosis or macropinocytosis did not affect PLA-NP uptake in the incubation times performed (Fig. 6a).

As flow cytometry analysis of PLA-NP exposure for 240 min indicated a reduction of PLA-NP cell uptake upon inhibition of lipid raft- and clathrin-mediated endocytosis, these conditions were further assessed by confocal microscopy that confirmed these results (Fig. 6b).

It has previously been demonstrated that treatment of cells with MCD, which depletes cholesterol components of the cell membrane, can not only influence lipid raft mediated uptake, but also clathrin-mediated endocytosis [65]. Therefore, the reduction in PLA-NP uptake observed in response to MCD shown here may actually infer an association with clathrin-mediated endocytosis (Fig. 5). However, the possibility that lipid rafts may be involved in PLA-NP uptake at these longer exposure times should not be discarded and deserves careful consideration. We observed co-localization of the endocytic cargo (PLA-NP) with ganglioside GM1-bound cholera toxin subunit B, which may provide some evidence for the involvement of lipid-raft mediated mechanisms. (Fig. 6c).

## Discussion

Drug delivery systems have been studied extensively to improve the efficacy of therapeutic products. Nanostructured materials attract strong interest for drug delivery due to advantages such as increased drug bioavailability and stability, drug targeting leading to a reduction of required administration dose and thus side effects, enhanced delivery of compounds with low water solubility, among others. Since metallic NP such as Au-NP, the most commonly used NP for drug delivery, have shown potential to induce cytotoxicity [9, 14, 15, 37], PLA-NP emerged as a viable alternative due to their biocompatible and biodegradable characteristics and the potential to control drug release kinetics [43, 63]. In fact it is often Au-NP functionalization which can be implicit in the biological responses that they elicit [9, 57]. Therefore, this study aimed to perform a detailed evaluation of PLA-NP induced cell death and stress responses, as well as the mechanisms involved in their internalization in human lung epithelial cells.

The presented results indicated that PLA-NP were stable over time. The observed difference of diameter between TEM and DLS may be attributed to the formation of a protein corona due to PLA-NP interaction with cell culture medium [34, 45], and/or water shell surrounding the NP [25]. As expected, the widely used methods for analysis of cell toxicity such as MTT conversion to formazan, LDH release and intracellular ATP levels did not show loss of viability in A549 cells treated with PLA-NP. It is important to emphasize that no NP interference was observed in these methods. In addition, the label-free real-time electrical impedance showed neither reduced viability nor alterations to cell proliferation, in response to PLA-NPs. Altogether, these data are in accordance with previous studies showing that PLA-NP did not affect the viability of HEK293, CHO-K1, MCF-7, SK-BR-3 or retinal pigment epithelium cells measured through the analysis of formazan reaction products [6, 7, 43, 46].

Notably, this study shows that IL-12 and VEGF levels were reduced upon 24 h PLA-NP exposure, returning to background levels by 48 h. IL-12 is related to inhibition of lung adenocarcinoma cell migration and invasion [39] and protective immunity against lung infection mediated by methicillin-resistant *Staphylococcus aureus* (MRSA), a common pathogen related to pneumonia [49]. Despite data demonstrating that PLA-NP did not affect A549 cell viability and proliferation, reduced levels of IL-12 during 24 h exposures suggest that the use of PLA-NP as a nanocarrier may contribute to increased vulnerability of lung epithelial cells to opportunistic infections. Regarding VEGF, it is involved with angiogenesis, growth and proliferation of lung epithelial cells through autocrine mechanisms, as well as in tissue repair and survival [27, 30, 48]. In addition, reduced levels of VEGF result in increased markers of oxidative stress and structural alterations of alveoli [72], being implicated in the pathogenesis of restrictive and obstructive lung diseases such as emphysema [74]. Nevertheless, it is conceivable that the transient reduction of IL-12 and VEGF levels induced by PLA-NP may not impact on lung epithelial cell function. Moreover, other studies have shown that A549 cells produce pro-inflammatory cytokines upon NP exposure. Titanium dioxide NP increased IL-2, IL-6, IFN-γ and TNF-α levels [44], while silver NP increased TNF-α levels [32]. In contrast, our results exposing A549 cells to PLA-NP did not induce these cytokines, reinforcing the biocompatibility of PLA-NP in A549 cells.

Proteomics data showed that expression of different HSPs, which are involved in stress responses and virus interaction, were prominently increased, more so than other proteins. HSPs are highly conserved molecular chaperones displaying key roles in signal transduction, protein folding and degradation. HSP90 is involved in the folding, assembly, maturation and stabilization of proteins, important to survival and proliferation [19]. Members of the HSP90 family were upregulated in our analysis, including HSP90B2P, GRP94c and tumor rejection antigen Gp96 (TRA1). Studies have suggested that GRP94 upregulation occurs under hypoxia [55], and is involved in apoptosis when homeostasis of endoplasmic reticulum is disrupted [31]. In the context of endoplasmic reticulum stress, upregulation of hsp gene expression upon Au-NP exposure has previously been reported in *C. elegans* and a protective role for these gene products has been postulated [71].

Here, HSP70 and HSP60 chaperonin members (HSPA1A; HSPA5; HSPA6; HSPA8; HSPD1) were upregulated in A549 cells in response to PLA-NP. Studies have shown that HSPA1A and HSPA5 display protective properties to stress conditions, amyloid polypeptide aggregation and toxicity and apoptosis [58]. The expression of HSP70, HSP90, GRP78, and GRP94 is increased along hepatocarcinogenesis progression. GRP78 and GRP94 are related to vascular invasion and intrahepatic metastasis [40]. Moreover, HSPA8 and HSPD1 chaperonins are related to host-virus interaction in biological processes. HSPA8 mediates an LPS-induced inflammatory response, including TNF-α secretion by monocytes [70]. HSPD1 is able to interact with interferon regulatory factor 3 (IRF3) contributing to IFN-β signaling during Sendai virus infection [41].

The potential role of HSP22, HSP60, HSP70, and HSP72 to act as danger-associated molecular patterns (DAMPs) in pro-inflammatory responses leading to activation of innate immune cells including neutrophils, macrophages, monocytes, and dendritic cells through their interaction with Toll-like receptors (TLRs)-2 and -4, has been extensively investigated [3, 13]. Hence, the increased expression of HSPs in response to PLA-NP may infer promotion of cell survival mechanisms, in which the stress responses observed within the cells’ proteome occurred in the absence of cell death or loss of cell viability, as demonstrated in the assays based on metabolic activity (MTT, ATP) and membrane integrity (LDH). One limitation of the proteomic results described here is that only a single exposure condition (one NP concentration and time point) was tested, therefore, this preliminary data on a potential upregulation of HSPs upon PLA-NP treatment require further validation covering a more comprehensive range of time points and NP concentrations.

**Fig. 7:**
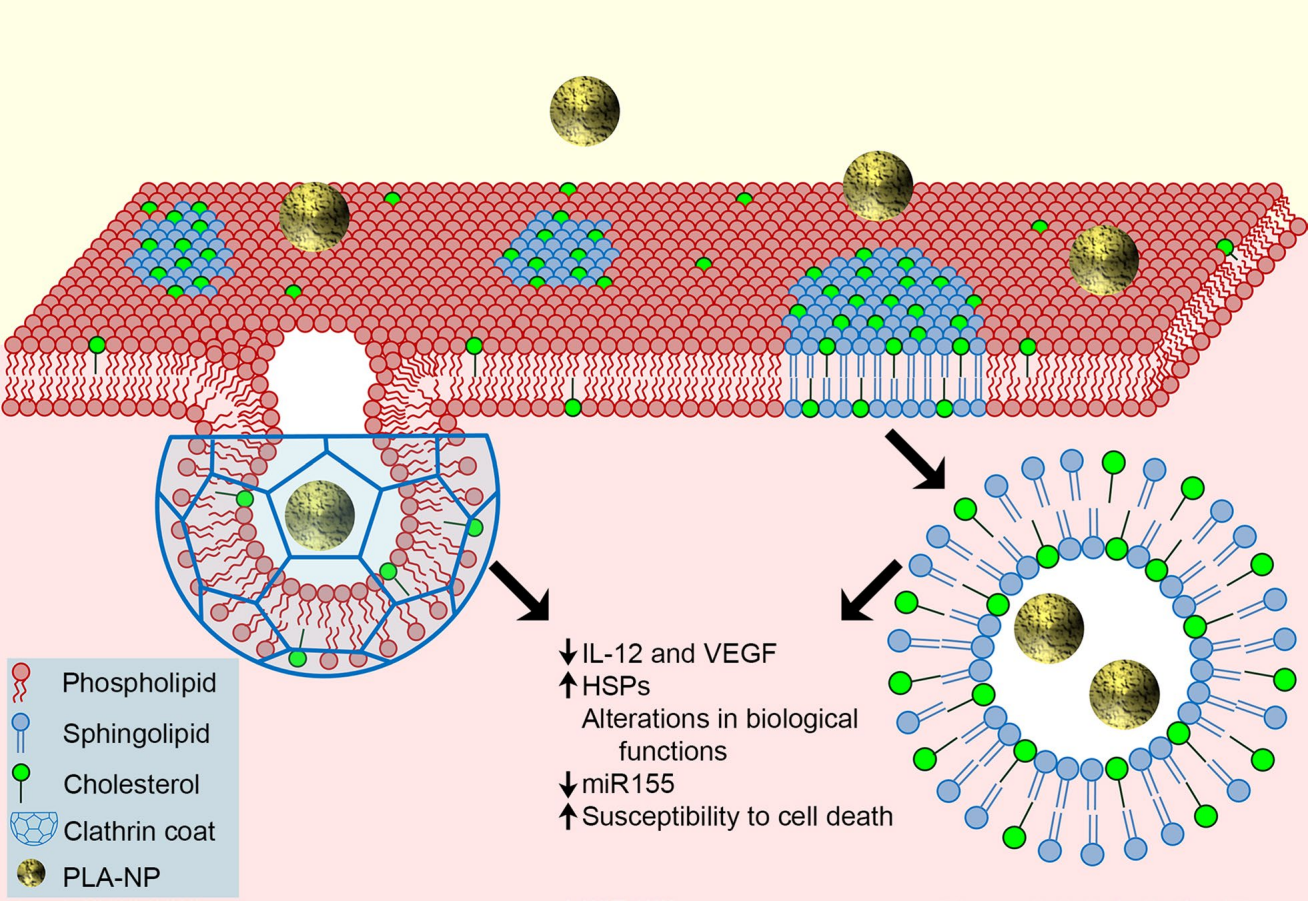
Schematic representation of PLA-NP internalization through clathrin-coated pits and lipid rafts in cell membrane. Endocytosis mediated by clathrin-coated pits is a fast and dynamic mechanism beginning with membrane invagination. This process involves different stages such as initiation, growth, stabilization and pit commitment, cargo capture, budding, scission and uncoating, during ~45 to ~80 s. This mechanism is dependent on proteins such as clathrin and dynamin. The diameter of coated vesicles vary from ~70 to ~135 nm, according to cell type [33]. Lipid rafts are microdomains enriched in cholesterol and sphingolipids such as sphingomyelin. Such membrane specialization is a less fluid region that compartmentalizes proteins related to intracellular signaling pathways. Planar lipid rafts can be associated to flotillins mediating endocytosis in a clathrin- and caveolin-independent manner by a still unknown mechanism [47]. In this work, we observed that PLA-NP uptake was mediated preponderantly by clathrin-coated pits and lipid rafts in A549 cells. Despite the non-alteration in cell viability, PLA-NP promoted modifications in biological functions that may affect cell physiology. IL-12 and VEGF secretion and miR155 levels were reduced while intracellular HSPs levels were increased. Such alterations may contribute to increased susceptibility to cell death

Due to the absence of cytotoxicity, while alterations to intracellular proteins involved in cell functions was evident, we investigated whether PLA-NP could trigger gene alterations related to inflammation and cell death processes, through determination of mRNA and miRNA. While no change was observed for mRNA, miR155 was strongly reduced. Distinct expression profiles of miR155 seem to play a role in both physiological and pathological processes such as immunity, inflammation, hematopoiesis, and cardiovascular and neoplastic diseases [21]. Indeed, some miRNAs, including miR155, are increased in human lung cancer and it has been suggested as a biomarker of poor prognosis in lung cancer patients [59, 76]. The tumor suppressor TP53 mRNA is a potential miR155 target. However, in the present study, the expression levels of TP53 mRNA were evaluated and no changes were observed when comparing PLA-treated with untreated cells. Moreover, a study using A549 cells demonstrated that miR155 negatively regulates Apaf-1, a protein involved in apoptotic mechanisms, conferring cellular resistance to cisplatin treatment [78]. In this sense, this study evaluated whether reduced miR155 expression levels induced by PLA-NP could confer susceptibility to death stimuli in A549 cells. This hypothesis was reinforced by an increase in susceptibility of A549 cells to STS-induced cell death, when pre-stimulated with PLA-NP. In the context of lung infection, PLA-NP may influence inflammatory responses and increase cell death. Our hypothesis is supported by a study showing that previous acute exposure to silica NP increased the susceptibility of mice to lethal pneumonia induced by *Pseudomonas aeruginosa* [16]. Such results suggest that chronic use of PLA-NP requires attention to these markers (Fig. 7).

The endocytic mechanisms related to PLA-NP uptake in A549 cells were also investigated. The results showed that such NP are internalized mostly by clathrin-mediated endocytosis or in association with lipid rafts. PLA-NP endocytosis mediated by clathrin-coated pits occurred within 60 min, where the major levels of internalization may be attributed to a quick mechanism ranging from ~45 to ~80 s. This involves different stages such as initiation, growth, stabilization and pit commitment cargo capture, budding and scission, and finally uncoating [33]. Previously, it was suggested that poly(lactic-co-glycolic acid) NP (PLGA-NP) and solid lipid NP uptake occurred mainly through a clathrin-dependent manner in lung epithelial A549 cells, using hypertonic sucrose medium [61, 67, 68]. However, it is well known that hypertonic sucrose medium also inhibits the uptake of cholera toxin, a lipid raft ligand [12]. Moreover, hypertonic sucrose medium inhibits endocytosis mediated by pinocytosis [10, 66]. These studies strongly support that the use of hypertonic sucrose medium is not selective to inhibit clathrin-coated pits. In this sense, lipid rafts could also be involved in NP uptake. In the present work, a competitive inhibitor of clathrin terminal domains was used, which selectively inhibits clathrin-mediated endocytosis. Thus, it was possible to infer that clathrin pits are involved in PLA-NP uptake in lung epithelial A549 cells (Fig. 7).

The endocytosis of PLA-NP mediated by lipid rafts was observed after 240 min exposure through cholesterol depletion. However, clathrin- and caveolae-mediated endocytosis can also be sensitive to cholesterol depletion [11, 65, 79]. In this respect, it is possible that the late effect we observed in response to MCD may be attributed to inhibition of clathrin pits. However, the intracellular co-localization of PLA-NP and ganglioside M1 prompted us to infer that PLA-NP are taken up also through lipid rafts. In fact, studies have demonstrated that planar lipid rafts can be associated to flotillins mediating endocytosis in a clathrin- and caveolin-independent manner [23, 26, 53]. No involvement of caveolae-mediated uptake mechanisms was considered in the PLA-NP used in this study.

Previous studies have shown that the majority of NP administrated through inhalation are phagocytized by alveolar macrophages [24, 64]. Upon internalization of PLA-NP, lysosomes are considered as their intracellular fate [5]. Moreover, it is important to emphasize that the uptake mechanisms are also dependent on physicochemical properties of the NP, such as material composition, size, shape and others, as well as the cell type investigated, as has been reviewed recently [62]. In the present study, PLA-NP internalization in A549 cells mediated by clathrin-coated pits appeared to be dominant over other endocytic pathways in initial cell contact, but lipid rafts display relevant involvement in extended periods of PLA-NP incubation. These results may help to improve strategies aiming for selective nanocarrier uptake through proteins/biomarkers located in membrane structures involved in endocytosis.

## Conclusions

Using high-throughput screening methods and more complex methodologies, we have demonstrated that the biodegradable PLA-NP induced alterations to the proteome of the A549 lung epithelial cell line. PLA-NP strongly increased HSPs proteins related to cell stress and decreased the secretion of soluble mediators and miR155 levels. Such critical modifications in cell physiology may indicate increased susceptibility to apoptosis. Moreover, PLA-NP uptake was mostly by clathrin-dependent mechanisms with lipid raft involvement during long periods of incubation. These data can contribute to the development of safe-by-design nanocarriers and to the improvement of selective uptake strategies.

## Additional files

**Additional file 1: Table S1.** Upregulated polypeptides identified by mass spectrometry from A549 proteome in response to PLA-NP with their respective description.

**Additional file 2: Table S2.** Downregulated polypeptides identified by mass spectrometry from A549 proteome in response to PLA-NP with their respective description.

## Abbreviations

ATP: adenosine triphosphate
Au-NP: gold nanoparticles
CCR5: C–C chemokine receptor type 5
DAMP: danger-associated molecular patterns
Dapi: 4′,6-diamidino-2-phenylindole
DLS: dynamic light scattering
DMSO: dimethyl sulfoxide
DTT: dithiothreitol
FACS: flow cytometry
FBS: fetal bovine serum
FDA: Food and Drug Administration
GO: Gene Ontology
HSP: heat shock protein
IAA: iodoacetamide
IL: interleucine
IRF3: interferon regulatory factor 3
LC–MS/MS: liquid chromatography tandem mass spectrometry
LDH: lactate dehydrogenase
MCD: methyl-β-cyclodextrin
miRNA: micro-RNA
mRNA: messenger RNA
MRSA: methicillin-resistant *Staphylococcus aureus*
MSE: mass spectrometry mode
MTT: 3-(4,5-dimethyl-2-thiazolyl)-2,5-diphenyl-2H-tetrazolium bromide
PLA-NP: poly-lactic acid nanoparticles
PLGA-NP: poly-lactic-co-glycolic acid nanoparticles
qPCR: quantitative real-time polymerase chain reaction
RT: room temperature
STS: staurosporine
TEM: transmission electron microscopy
TLR: toll-like receptor
