# Supplementary material for: Poly-lactic acid nanoparticles (PLA-NP) promote physiological modifications in lung epithelial cells and are internalized by clathrin-coated pits and lipid rafts
Source: J Nanobiotechnology. 2017 Jan 31;15:11. doi: 10.1186/s12951-016-0238-1 (PMC5282631; doi:10.1186/s12951-016-0238-1)
Supplement: Supplementary file 1 — Additional file 1: Table S1. Upregulated polypeptides identified by mass spectrometry from A549 proteome in response to PLA-NP with their respective description. [file 12951_2016_238_MOESM1_ESM.docx]

| **Upregulated polypeptides identified by mass spectrometry from A549 proteome in response to PLA-NP** | | | | | | |
| --- | --- | --- | --- | --- | --- | --- |
| **Accession** | **Description** | **Id Score** | **Unique** | **G2/G1** | **GO Function** | **GO Category** |
| 1433F | 14 3 3 protein eta OS Homo sapiens GN YWHAH PE 1 SV 4 | 2474,03 | G2 | 4,66 | [Apoptotic process/ gene expression/ membrane organization](http://www.ebi.ac.uk/QuickGO/GTerm?id=GO:0006915) | Biological Process |
| A0JNT2 | KRT83 protein OS Homo sapiens GN KRT83 PE 2 SV 1 | 1055,53 | G2 | - | [Structural molecule activity](http://www.ebi.ac.uk/QuickGO/GTerm?id=GO:0005198) | Molecular Function |
| A1L0V1 | ACTN1 protein Fragment OS Homo sapiens GN ACTN1 PE 2 SV 1 | 2407,69 | G2 | - | [Actin crosslink formation / actin filament bundle assembly](http://www.ebi.ac.uk/QuickGO/GTerm?id=GO:0051764) | Biological Process |
| A1XP52 | Catecholamine regulated protein 40 OS Homo sapiens PE 2 SV 1 | 696,4 | G2 | - | [ATP binding](http://www.ebi.ac.uk/QuickGO/GTerm?id=GO:0005524) | Molecular Function |
| A4QMW8 | Enolase Fragment OS Homo sapiens GN ENO1 PE 2 SV 1 | 17654,11 | G2 | - | [Glycolysis](http://www.uniprot.org/keywords/KW-0324) | Biological Process |
| A8K9J7 | Histone H2B OS Homo sapiens PE 2 SV 1 | 8878,4 | G2 | - | [Chromatin organization / chromatin silencing](http://www.ebi.ac.uk/QuickGO/GTerm?id=GO:0006325) | Biological Process |
| A8MUS3 | 60S ribosomal protein L23a OS Homo sapiens GN RPL23A PE 1 SV 1 | 601,81 | G2 | - | [Translation](http://www.ebi.ac.uk/QuickGO/GTerm?id=GO:0006412) | Biological Process |
| ACTBM | Putative beta actin like protein 3 OS Homo sapiens GN POTEKP PE 5 SV 1 | 14021,91 |  | 1,17 | [Blood coagulation / platelet activation / platelet degranulation](http://www.ebi.ac.uk/QuickGO/GTerm?id=GO:0007596) | Biological Process |
| ACTC | Actin alpha cardiac muscle 1 OS Homo sapiens GN ACTC1 PE 1 SV 1 | 69282,63 | G2 | - | [Actin filament-based movement / apoptotic process / response to drug](http://www.ebi.ac.uk/QuickGO/GTerm?id=GO:0030048) | Biological Process |
| ACTS | Actin alpha skeletal muscle OS Homo sapiens GN ACTA1 PE 1 SV 1 | 69282,63 | G2 | - | [Cell growth / response to extracellular stimulus / mesenchyme migration](http://www.ebi.ac.uk/QuickGO/GTerm?id=GO:0016049) | Biological Process |
| AINX | Alpha internexin OS Homo sapiens GN INA PE 1 SV 2 | 174,47 | G2 | - | Differentiation, Neurogenesis | Biological Process |
| AK1BA | Aldo keto reductase family 1 member B10 OS Homo sapiens GN AKR1B10 PE 1 SV 2 | 5483,65 |  | 1,11 | [Cellular aldehyde metabolic process / daunorubicin metabolic process / digestion / Oxidoreductase](http://www.ebi.ac.uk/QuickGO/GTerm?id=GO:0006081) | Biological Process |
| AL1B1 | Aldehyde dehydrogenase X mitochondrial OS Homo sapiens GN ALDH1B1 PE 1 SV 3 | 441,58 | G2 | 2,36 | [Carbohydrate metabolic process / ethanol catabolic process / ethanol oxidation / small molecule metabolic process / xenobiotic metabolic process](http://www.ebi.ac.uk/QuickGO/GTerm?id=GO:0005975) | Biological Process |
| ALDOA | Fructose bisphosphate aldolase A OS Homo sapiens GN ALDOA PE 1 SV 2 | 3079,68 | G2 | 1,06 | [Glycolysis](http://www.uniprot.org/keywords/KW-0324) | Biological Process |
| ALDOC | Fructose bisphosphate aldolase C OS Homo sapiens GN ALDOC PE 1 SV 2 | 261,86 | G2 | - | [Glycolysis](http://www.uniprot.org/keywords/KW-0324) | Biological Process |
| ATPA | ATP synthase subunit alpha mitochondrial OS Homo sapiens GN ATP5A1 PE 1 SV 1 | 1189,07 | G2 | - | ATP synthesis, Hydrogen ion transport | Biological Process |
| ATPB | ATP synthase subunit beta mitochondrial OS Homo sapiens GN ATP5B PE 1 SV 3 | 1112,27 |  | 1,62 | ATP synthesis, Hydrogen ion transport | Biological Process |
| B3KQT9 | Protein disulfide isomerase OS Homo sapiens PE 2 SV 1 | 1323,02 | G2 | 1,3 | [Cell redox homeostasis](http://www.ebi.ac.uk/QuickGO/GTerm?id=GO:0045454) | Biological Process |
| B4DLR8 | NAD P H dehydrogenase quinone 1 OS Homo sapiens GN NQO1 PE 1 SV 1 | 3610,19 | G2 | 1,43 | None | None |
| B4DNH8 | Annexin OS Homo sapiens PE 2 SV 1 | 3224,93 | G2 | G2 | [Calcium-dependent phospholipid binding / calcium ion binding / phospholipase inhibitor activity](http://www.ebi.ac.uk/QuickGO/GTerm?id=GO:0005544) | Molecular function |
| B4DNW7 | Adenylyl cyclase associated protein OS Homo sapiens PE 2 SV 1 | 644,83 | G2 | G2 | [Actin cytoskeleton organization / cell morphogenesis](http://www.ebi.ac.uk/QuickGO/GTerm?id=GO:0030036) | Biological Process |
| B4DSU6 | Heterogeneous nuclear ribonucleoproteins C1 C2 OS Homo sapiens GN HNRNPC PE 1 SV 1 | 1735,32 | G2 | G2 | [Nucleic acid binding / nucleotide binding /Ribonucleoprotein](http://www.ebi.ac.uk/QuickGO/GTerm?id=GO:0003676) | Molecular function |
| B4DUZ8 | Adenylyl cyclase associated protein OS Homo sapiens PE 2 SV 1 | 584,38 | G2 | G2 | [Actin cytoskeleton organization / cell morphogenesis](http://www.ebi.ac.uk/QuickGO/GTerm?id=GO:0030036) | Biological Process |
| B5BU24 | 14 3 3 protein beta alpha OS Homo sapiens GN YWHAB PE 2 SV 1 | 682,52 | G2 | G2 | None | None |
| B5BU38 | Annexin OS Homo sapiens GN ANXA1 PE 2 SV 1 | 34339,52 | G2 | 1,09 | [Calcium-dependent phospholipid binding / calcium ion binding / phospholipase inhibitor activity](http://www.ebi.ac.uk/QuickGO/GTerm?id=GO:0005544) | Molecular function |
| B7Z3K9 | Fructose bisphosphate aldolase OS Homo sapiens PE 2 SV 1 | 261,86 | G2 | G2 | [Glycolysis](http://www.uniprot.org/keywords/KW-0324) | Biological Process |
| B8ZZ54 | 10 kDa heat shock protein mitochondrial OS Homo sapiens GN HSPE1 PE 1 SV 1 | 25215,78 | G2 | G2 | [Stress response](http://www.uniprot.org/keywords/KW-0346) | Biological Process |
| B9VP19 | 60 kDa chaperonin Fragment OS Homo sapiens GN HSPD1 PE 4 SV 1 | 2421,07 | G2 | G2 | [Host-virus interaction](http://www.uniprot.org/keywords/KW-0945) | Biological Process |
| C9J9K3 | 40S ribosomal protein SA Fragment OS Homo sapiens GN RPSA PE 1 SV 3 | 1084,01 |  | 1,13 | [rRNA export from nucleus / endonucleolytic cleavage in ITS1 to separate SSU-rRNA from 5.8S rRNA and LSU-rRNA from tricistronic rRNA transcript (SSU-rRNA, 5.8S rRNA, LSU-rRNA)](http://www.ebi.ac.uk/QuickGO/GTerm?id=GO:0006407) | Biological Process |
| C9JL25 | 60 kDa heat shock protein mitochondrial Fragment OS Homo sapiens GN HSPD1 PE 1 SV 1 | 11011,52 | G2 | 3 | [Host-virus interaction](http://www.uniprot.org/keywords/KW-0945) | Biological Process |
| CATD | Cathepsin D OS Homo sapiens GN CTSD PE 1 SV 1 | 286,63 | G2 | G2 | [Antigen processing and presentation of exogenous peptide antigen via MHC class II / autophagy / collagen catabolic process / extracellular matrix disassembly / extracellular matrix organization / protein catabolic process / proteolysis](http://www.ebi.ac.uk/QuickGO/GTerm?id=GO:0019886) | Biological Process |
| CH60 | 60 kDa heat shock protein mitochondrial OS Homo sapiens GN HSPD1 PE 1 SV 2 | 11357,17 |  | 1,13 | [Host-virus interaction](http://www.uniprot.org/keywords/KW-0945) | Biological Process |
| D3DPI2 | HCG1641229 isoform CRA a OS Homo sapiens GN hCG 1641229 PE 4 SV 1 | 1391,46 | G2 | G2 | [Nucleic acid binding / nucleotide binding](http://www.ebi.ac.uk/QuickGO/GTerm?id=GO:0003676) | Molecular function |
| D3GKD8 | A gamma globin Osilo variant OS Homo sapiens GN HBG1 PE 3 SV 1 | 14069,3 | G2 | G2 | [Oxygen transport](http://www.uniprot.org/keywords/KW-0561) | Biological Process |
| D6R9L0 | Guanine nucleotide binding protein subunit beta 2 like 1 Fragment OS Homo sapiens GN GNB2L1 PE 1 S | 422,87 | G2 | G2 | None | None |
| D6RE83 | Ubiquitin carboxyl terminal hydrolase OS Homo sapiens GN UCHL1 PE 1 SV 1 | 770,36 | G2 | G2 | [Ubl conjugation pathway](http://www.uniprot.org/keywords/KW-0833) | Biological Process |
| D6RHH4 | Guanine nucleotide binding protein subunit beta 2 like 1 OS Homo sapiens GN GNB2L1 PE 1 SV 1 | 1148,44 | G2 | G2 | None | None |
| D9YZU8 | Hemoglobin gamma A OS Homo sapiens GN HBG1 PE 3 SV 1 | 14069,3 | G2 | 4,06 | [Oxygen transport](http://www.uniprot.org/keywords/KW-0561) | Biological Process |
| E7ESH4 | 60 kDa heat shock protein mitochondrial Fragment OS Homo sapiens GN HSPD1 PE 1 SV 1 | 6078,47 | G2 | 1,54 | [ATP binding / Chaperone](http://www.ebi.ac.uk/QuickGO/GTerm?id=GO:0005524) | Molecular function |
| E9PBW4 | Hemoglobin subunit gamma 2 OS Homo sapiens GN HBG2 PE 1 SV 1 | 14055,19 | G2 | G2 | [Oxygen transport](http://www.uniprot.org/keywords/KW-0561) | Biological Process |
| E9PCY7 | Heterogeneous nuclear ribonucleoprotein H OS Homo sapiens GN HNRNPH1 PE 1 SV 1 | 671,88 | G2 | 1,88 | mRNA processing, mRNA splicing | Biological Process |
| E9PFF2 | Transketolase OS Homo sapiens GN TKT PE 1 SV 1 | 7461,09 | G2 | 5,53 | None | None |
| E9PK86 | Serpin H1 Fragment OS Homo sapiens GN SERPINH1 PE 1 SV 1 | 551,03 | G2 | G2 | [Stress response](http://www.ebi.ac.uk/QuickGO/GTerm?id=GO:0030199) | Biological Process |
| ENOA | Alpha enolase OS Homo sapiens GN ENO1 PE 1 SV 2 | 14461,99 |  | 1,15 | Glycolysis, Plasminogen activation, Transcription, Transcription regulation | Biological Process |
| ENPLL | Putative endoplasmin like protein OS Homo sapiens GN HSP90B2P PE 5 SV 1 | 84,6 | G2 | G2 | [Stress response](http://www.uniprot.org/keywords/KW-0346) | Biological Process |
| EZRI | Ezrin OS Homo sapiens GN EZR PE 1 SV 4 | 208,3 | G2 | G2 | [Cell shape](http://www.uniprot.org/keywords/KW-0133) | Biological Process |
| F2Z2Y4 | Pyridoxal kinase OS Homo sapiens GN PDXK PE 1 SV 1 | 326,55 | G2 | G2 | [Pyridoxal 5'-phosphate salvage](http://www.ebi.ac.uk/QuickGO/GTerm?id=GO:0009443) | Biological Process |
| F2Z393 | Transaldolase OS Homo sapiens GN TALDO1 PE 1 SV 1 | 305,7 | G2 | G2 | [Pentose shunt](http://www.uniprot.org/keywords/KW-0570) | Biological Process |
| F5H018 | GTP binding nuclear protein Ran Fragment OS Homo sapiens GN RAN PE 1 SV 3 | 1936,91 | G2 | G2 | [Protein import into nucleus / small GTPase mediated signal transduction](http://www.ebi.ac.uk/QuickGO/GTerm?id=GO:0006606) | Biological Process |
| F5H245 | L lactate dehydrogenase OS Homo sapiens GN LDHC PE 1 SV 1 | 464,86 | G2 | G2 | [Carbohydrate metabolic process (carboxylic acid metabolic process)](http://www.ebi.ac.uk/QuickGO/GTerm?id=GO:0005975) | Biological Process |
| F8VPE8 | 60S acidic ribosomal protein P0 Fragment OS Homo sapiens GN RPLP0 PE 1 SV 1 | 971,14 | G2 | G2 | [Ribosome biogenesis](http://www.ebi.ac.uk/QuickGO/GTerm?id=GO:0042254) | Biological Process |
| F8VY02 | Endoplasmic reticulum resident protein 29 OS Homo sapiens GN ERP29 PE 1 SV 1 | 2531,59 | G2 | 1,39 | [Protein secretion](http://www.ebi.ac.uk/QuickGO/GTerm?id=GO:0009306) | Biological Process |
| F8W079 | ATP synthase subunit beta mitochondrial Fragment OS Homo sapiens GN ATP5B PE 1 SV 1 | 172,63 | G2 | G2 | [Hydrogen ion transport](http://www.uniprot.org/keywords/KW-0375) | Biological Process |
| FABP5 | Fatty acid binding protein epidermal OS Homo sapiens GN FABP5 PE 1 SV 3 | 599,49 | G2 | G2 | [Transport](http://www.uniprot.org/keywords/KW-0813) | Biological Process |
| G3P | Glyceraldehyde 3 phosphate dehydrogenase OS Homo sapiens GN GAPDH PE 1 SV 3 | 30228 |  | 1,68 | Apoptosis, Glycolysis, Translation regulation | Biological Process |
| G3V1A4 | Cofilin 1 Non muscle isoform CRA a OS Homo sapiens GN CFL1 PE 1 SV 1 | 12004,3 | G2 | G2 | [Actin filament depolymerization](http://www.ebi.ac.uk/QuickGO/GTerm?id=GO:0030042) | Biological Process |
| G3V3R4 | HCG1983504 isoform CRA c OS Homo sapiens GN TUBB3 PE 1 SV 1 | 5013,89 | G2 | 2,29 | [Microtubule-based process](http://www.ebi.ac.uk/QuickGO/GTerm?id=GO:0007017) | Biological Process |
| GRP78 | 78 kDa glucose regulated protein OS Homo sapiens GN HSPA5 PE 1 SV 2 | 2929,88 |  | 1,31 | [Blood coagulation /cellular response to interleukin-4 / PERK-mediated unfolded protein response](http://www.ebi.ac.uk/QuickGO/GTerm?id=GO:0007596) | Biological Process |
| H0YA55 | Serum albumin Fragment OS Homo sapiens GN ALB PE 1 SV 1 | 2743,51 | G2 | G2 | Transport | Biological Process |
| H0YK49 | Electron transfer flavoprotein subunit alpha mitochondrial OS Homo sapiens GN ETFA PE 1 SV 1 | 326,61 | G2 | G2 | [Electron carrier activity / flavin adenine dinucleotide binding](http://www.ebi.ac.uk/QuickGO/GTerm?id=GO:0009055) | Molecular function |
| H0YLV6 | Annexin OS Homo sapiens GN ANXA2 PE 1 SV 1 | 5135,38 | G2 | G2 | [Calcium-dependent phospholipid binding / calcium ion binding / phospholipase inhibitor activity](http://www.ebi.ac.uk/QuickGO/GTerm?id=GO:0005544) | Molecular function |
| H0YNP5 | Annexin Fragment OS Homo sapiens GN ANXA2 PE 1 SV 1 | 525,5 | G2 | G2 | Calcium-dependent phospholipid binding / calcium ion binding / phospholipase inhibitor activity | Molecular function |
| H2A1A | Histone H2A type 1 A OS Homo sapiens GN HIST1H2AA PE 1 SV 3 | 27883,63 |  | 1,42 | [Chromatin organization / chromatin silencing](http://www.ebi.ac.uk/QuickGO/GTerm?id=GO:0006325) | Biological Process |
| H2B3B | Histone H2B type 3 B OS Homo sapiens GN HIST3H2BB PE 1 SV 3 | 17277,43 | G2 | 1,49 | [Chromatin organization / nucleosome assembly](http://www.ebi.ac.uk/QuickGO/GTerm?id=GO:0006325) | Biological Process |
| H31T | Histone H3 1t OS Homo sapiens GN HIST3H3 PE 1 SV 3 | 2531,49 | G2 | 1,54 | [Nucleosome assembly](http://www.ebi.ac.uk/QuickGO/GTerm?id=GO:0006281) | Biological Process |
| H3BR70 | Pyruvate kinase OS Homo sapiens GN PKM PE 1 SV 1 | 4655,95 | G2 | G2 | [Glycolysis](http://www.uniprot.org/keywords/KW-0324) | Biological Process |
| H4 | Histone H4 OS Homo sapiens GN HIST1H4A PE 1 SV 2 | 7875,97 |  | 2,27 | [Nucleosome assembly](http://www.ebi.ac.uk/QuickGO/GTerm?id=GO:0044267) | Biological Process |
| H7C5W5 | Peripherin Fragment OS Homo sapiens GN PRPH PE 3 SV 1 | 545,12 | G2 | G2 | [Structural molecule activity](http://www.ebi.ac.uk/QuickGO/GTerm?id=GO:0005198) | Molecular function |
| H9KV75 | Alpha actinin 1 OS Homo sapiens GN ACTN1 PE 1 SV 1 | 4427,44 | G2 | G2 | [Actin crosslink formation / actin filament bundle assembly](http://www.ebi.ac.uk/QuickGO/GTerm?id=GO:0051764) | Biological Process |
| HAUS4 | HAUS augmin like complex subunit 4 OS Homo sapiens GN HAUS4 PE 1 SV 1 | 108,56 | G2 | 1,15 | Cell cycle/ Mitosis | Biological Process |
| HBD | Hemoglobin subunit delta OS Homo sapiens GN HBD PE 1 SV 2 | 14055,19 | G2 | G2 | Oxygen transport | Biological Process |
| HNRH1 | Heterogeneous nuclear ribonucleoprotein H OS Homo sapiens GN HNRNPH1 PE 1 SV 4 | 658,84 | G2 | 1,73 | mRNA processing, mRNA splicing | Biological Process |
| HNRH2 | Heterogeneous nuclear ribonucleoprotein H2 OS Homo sapiens GN HNRNPH2 PE 1 SV 1 | 118,29 | G2 | G2 | [mRNA processing, mRNA splicing (via spliceosome)](http://www.ebi.ac.uk/QuickGO/GTerm?id=GO:0010467) | Biological Process |
| HSP71 | Heat shock 70 kDa protein 1A 1B OS Homo sapiens GN HSPA1A PE 1 SV 5 | 12744,24 | G2 | G2 | [Stress response](http://www.uniprot.org/keywords/KW-0346) | Biological Process |
| HSP7C | Heat shock cognate 71 kDa protein OS Homo sapiens GN HSPA8 PE 1 SV 1 | 11060,33 |  | 1,28 | Host-virus interaction | Biological Process |
| I1VZV6 | Hemoglobin alpha 1 OS Homo sapiens GN HBA1 PE 3 SV 1 | 2941,85 | G2 | G2 | [Oxygen transport](http://www.uniprot.org/keywords/KW-0561) | Biological Process |
| K2C6B | Keratin type II cytoskeletal 6B OS Homo sapiens GN KRT6B PE 1 SV 5 | 276,55 | G2 | 5,21 | [Ectoderm development](http://www.ebi.ac.uk/QuickGO/GTerm?id=GO:0007398) | Biological Process |
| K2C7 | Keratin type II cytoskeletal 7 OS Homo sapiens GN KRT7 PE 1 SV 5 | 289,89 |  | 1,79 | [Host-virus interaction](http://www.uniprot.org/keywords/KW-0945) | Biological Process |
| K2C75 | Keratin type II cytoskeletal 75 OS Homo sapiens GN KRT75 PE 1 SV 2 | 186,52 |  | 2,34 | [Host-virus interaction](http://www.uniprot.org/keywords/KW-0945) | Biological Process |
| K2C8 | Keratin type II cytoskeletal 8 OS Homo sapiens GN KRT8 PE 1 SV 7 | 5191 |  | 1,8 | [Host-virus interaction](http://www.uniprot.org/keywords/KW-0945) | Biological Process |
| K7EKH9 | Glial fibrillary acidic protein Fragment OS Homo sapiens GN GFAP PE 4 SV 1 | 171,19 | G2 | G2 | [Structural molecule activity](http://www.ebi.ac.uk/QuickGO/GTerm?id=GO:0005198) | Molecular function |
| KRT86 | Keratin type II cuticular Hb6 OS Homo sapiens GN KRT86 PE 1 SV 1 | 187,91 | G2 | G2 | [Structural molecule activity](http://www.ebi.ac.uk/QuickGO/GTerm?id=GO:0005198) | Molecular function |
| L0R849 | Alternative protein EDARADD OS Homo sapiens GN EDARADD PE 3 SV 1 | 100,05 | G2 | 1,28 | [Glycolytic process](http://www.ebi.ac.uk/QuickGO/GTerm?id=GO:0006096) | Biological Process |
| LDHB | L lactate dehydrogenase B chain OS Homo sapiens GN LDHB PE 1 SV 2 | 4218,34 |  | 1,08 | [Carbohydrate metabolic process (lactate metabolic process)](http://www.ebi.ac.uk/QuickGO/GTerm?id=GO:0005975) | Biological Process |
| LRC45 | Leucine rich repeat containing protein 45 OS Homo sapiens GN LRRC45 PE 1 SV 1 | 104,95 | G2 | G2 | None | None |
| M0QZL7 | Tubulin beta 4A chain Fragment OS Homo sapiens GN TUBB4A PE 4 SV 3 | 1227,88 | G2 | G2 | [Microtubule-based process](http://www.ebi.ac.uk/QuickGO/GTerm?id=GO:0007017) | Biological Process |
| M0R1M6 | Ubiquitin 60S ribosomal protein L40 Fragment OS Homo sapiens GN UBA52 PE 1 SV 1 | 746,09 | G2 | G2 | [Translation](http://www.ebi.ac.uk/QuickGO/GTerm?id=GO:0006412) | Biological Process |
| NACAM | Nascent polypeptide associated complex subunit alpha muscle specific form OS Homo sapiens GN NACA P | 519,97 | G2 | 1,3 | Transcription, Transcription regulation | Biological Process |
| NUCL | Nucleolin OS Homo sapiens GN NCL PE 1 SV 3 | 420,23 | G2 | 1,15 | Angiogenesis / cellular response to lipopolysaccharide / endocytosis / liver regeneration / negative regulation of apoptotic process / positive regulation of interleukin-6 secretion / positive regulation of transcription from RNA polymerase II promoter / positive regulation of transcription of nuclear large rRNA transcript from RNA polymerase I promoter / positive regulation of tumor necrosis factor production / regulation of rRNA processing / spermatogenesis | Biological Process |
| PAL4D | Peptidyl prolyl cis trans isomerase A like 4D OS Homo sapiens GN PPIAL4D PE 3 SV 1 | 3124,49 | G2 | G2 | [Protein folding](http://www.ebi.ac.uk/QuickGO/GTerm?id=GO:0006457) | Biological Process |
| PDIA1 | Protein disulfide isomerase OS Homo sapiens GN P4HB PE 1 SV 3 | 504,68 | G2 | G2 | [Cell redox homeostasis / cellular response to hypoxia / extracellular matrix organization / proteolysis / regulation of oxidative stress-induced intrinsic apoptotic signaling pathway / response to endoplasmic reticulum stress / response to reactive oxygen species](http://www.ebi.ac.uk/QuickGO/GTerm?id=GO:0045454) | Biological Process |
| PGK1 | Phosphoglycerate kinase 1 OS Homo sapiens GN PGK1 PE 1 SV 3 | 1457,49 |  | 1,13 | [Glycolysis](http://www.uniprot.org/keywords/KW-0324) | Biological Process |
| POTEE | POTE ankyrin domain family member E OS Homo sapiens GN POTEE PE 1 SV 3 | 23959,43 |  | 1,68 | [Retina homeostasis](http://www.ebi.ac.uk/QuickGO/GTerm?id=GO:0001895) | Biological Process |
| POTEF | POTE ankyrin domain family member F OS Homo sapiens GN POTEF PE 1 SV 2 | 21761,07 |  | 1,27 | [Retina homeostasis](http://www.ebi.ac.uk/QuickGO/GTerm?id=GO:0001895) | Biological Process |
| POTEJ | POTE ankyrin domain family member J OS Homo sapiens GN POTEJ PE 3 SV 1 | 20842,77 |  | 11,36 | [Retina homeostasis](http://www.ebi.ac.uk/QuickGO/GTerm?id=GO:0001895) | Biological Process |
| PPIA | Peptidyl prolyl cis trans isomerase A OS Homo sapiens GN PPIA PE 1 SV 2 | 5831,44 |  | 1,19 | [Host-virus interaction](http://www.uniprot.org/keywords/KW-0945) | Biological Process |
| PRDX1 | Peroxiredoxin 1 OS Homo sapiens GN PRDX1 PE 1 SV 1 | 7671,38 |  | 1,13 | [Cell proliferation / erythrocyte homeostasis / hydrogen peroxide catabolic process / natural killer cell mediated cytotoxicity / regulation of NF-kappaB import into nucleus / regulation of stress-activated MAPK cascade / removal of superoxide radicals / response to reactive oxygen species](http://www.ebi.ac.uk/QuickGO/GTerm?id=GO:0008283) | Biological Process |
| PRDX5 | Peroxiredoxin 5 mitochondrial OS Homo sapiens GN PRDX5 PE 1 SV 4 | 338,67 | G2 | 3,46 | [Apoptotic process / cellular response to reactive oxygen species / inflammatory response / negative regulation of apoptotic process / negative regulation of cysteine-type endopeptidase activity involved in apoptotic process / regulation of apoptosis involved in tissue homeostasis](http://www.ebi.ac.uk/QuickGO/GTerm?id=GO:0006915) | Biological Process |
| PTBP1 | Polypyrimidine tract binding protein 1 OS Homo sapiens GN PTBP1 PE 1 SV 1 | 288,41 | G2 | 1,7 | mRNA processing, mRNA splicing | Biological Process |
| Q13707 | ACTA2 protein Fragment OS Homo sapiens GN ACTA2 PE 3 SV 1 | 55008,8 |  | 6,82 | None | None |
| Q2TSD0 | Glyceraldehyde 3 phosphate dehydrogenase OS Homo sapiens PE 2 SV 1 | 27689,8 |  | 1,63 | [Glycolysis](http://www.uniprot.org/keywords/KW-0324) | Biological Process |
| Q32Q12 | Nucleoside diphosphate kinase OS Homo sapiens GN NME1 NME2 PE 1 SV 1 | 2024,02 | G2 | 1,12 | [CTP biosynthetic process / GTP biosynthetic process / purine nucleotide metabolic process / pyrimidine nucleotide metabolic process / UTP biosynthetic process](http://www.ebi.ac.uk/QuickGO/GTerm?id=GO:0006241) | Biological Process |
| Q45VM7 | Mutant desmin OS Homo sapiens PE 2 SV 1 | 46,93 | G2 | G2 | [Structural molecule activity](http://www.ebi.ac.uk/QuickGO/GTerm?id=GO:0005198) | Molecular function |
| Q53FC7 | Heat shock 70kDa protein 6 HSP70B variant Fragment OS Homo sapiens PE 1 SV 1 | 7695,44 | G2 | 1,68 | [Stress response](http://www.uniprot.org/keywords/KW-0346) | Biological Process |
| Q53G35 | Phosphoglycerate mutase Fragment OS Homo sapiens PE 2 SV 1 | 1364,45 | G2 | G2 | [Glycolysis](http://www.uniprot.org/keywords/KW-0324) | Biological Process |
| Q53G99 | Beta actin variant Fragment OS Homo sapiens PE 2 SV 1 | 103768,8 | G2 | 24,53 | None | None |
| Q53HW2 | Ribosomal protein P0 variant Fragment OS Homo sapiens PE 2 SV 1 | 1414,47 | G2 | G2 | [Ribosome biogenesis](http://www.ebi.ac.uk/QuickGO/GTerm?id=GO:0042254) | Biological Process |
| Q562N4 | Actin like protein Fragment OS Homo sapiens GN ACT PE 3 SV 1 | 27939,04 | G2 | G2 | None | None |
| Q562P0 | Actin like protein Fragment OS Homo sapiens GN ACT PE 3 SV 1 | 17921,31 | G2 | G2 | None | None |
| Q562R8 | Actin like protein Fragment OS Homo sapiens GN ACT PE 3 SV 1 | 16762,67 | G2 | G2 | None | None |
| Q562X9 | Actin like protein Fragment OS Homo sapiens GN ACT PE 3 SV 1 | 18060,8 | G2 | G2 | None | None |
| Q58FF2 | Heat shock protein 94c OS Homo sapiens GN GRP94c PE 2 SV 1 | 116,14 | G2 | G2 | Stress response | Biological Process |
| Q59EQ2 | Tyrosine 3 monooxygenase tryptophan 5 monooxygenase activation protein beta polypeptide variant Fr | 1931,46 | G2 | G2 | [Monooxygenase activity](http://www.ebi.ac.uk/QuickGO/GTerm?id=GO:0004497) | Molecular function |
| Q59FS3 | Ribosomal protein S7 variant Fragment OS Homo sapiens PE 2 SV 1 | 1186,25 | G2 | G2 | [Translation](http://www.ebi.ac.uk/QuickGO/GTerm?id=GO:0006412) | Biological Process |
| Q59GR8 | TPM1 protein variant Fragment OS Homo sapiens PE 2 SV 1 | 2669,07 | G2 | 5,42 | None | None |
| Q59H49 | Polypyrimidine tract binding protein 1 isoform c variant Fragment OS Homo sapiens PE 2 SV 1 | 455,15 | G2 | G2 | [mRNA processing](http://www.ebi.ac.uk/QuickGO/GTerm?id=GO:0006397) | Biological Process |
| Q5CAQ5 | Tumor rejection antigen Gp96 1 OS Homo sapiens GN TRA1 PE 2 SV 1 | 1319,43 |  | 1,32 | [Response to stress](http://www.ebi.ac.uk/QuickGO/GTerm?id=GO:0006457) | Biological Process |
| Q5T6W5 | Heterogeneous nuclear ribonucleoprotein K OS Homo sapiens GN HNRNPK PE 1 SV 1 | 783,69 | G2 | 1,62 | Entry was delete | Entry was delete |
| Q5T8M8 | Actin alpha skeletal muscle OS Homo sapiens GN ACTA1 PE 3 SV 1 | 67143,52 | G2 | 3,46 | Entry was delete | Entry was delete |
| Q5TEC6 | Histone H3 OS Homo sapiens GN HIST2H3PS2 PE 1 SV 1 | 695,32 | G2 | G2 | [DNA binding](http://www.ebi.ac.uk/QuickGO/GTerm?id=GO:0003677) | Molecular function |
| Q6NSF2 | RPLP0 protein OS Homo sapiens GN RPLP0 PE 2 SV 1 | 1097,43 | G2 | G2 | [Ribosome biogenesis](http://www.ebi.ac.uk/QuickGO/GTerm?id=GO:0042254) | Biological Process |
| Q6V0K9 | Mutant hemoglobin beta chain Fragment OS Homo sapiens GN HBB PE 3 SV 1 | 11794,57 | G2 | G2 | [Oxygen transport](http://www.uniprot.org/keywords/KW-0561) | Biological Process |
| Q70T18 | BBF2H7 FUS protein Fragment OS Homo sapiens PE 2 SV 1 | 382,44 | G2 | G2 | [Nucleic acid binding / nucleotide binding](http://www.ebi.ac.uk/QuickGO/GTerm?id=GO:0003676) | Molecular function |
| Q7KZ24 | Nuclease sensitive element binding protein 1 OS Homo sapiens PE 2 SV 1 | 1795,5 | G2 | G2 | [Regulation of transcription (DNA-templated)](http://www.ebi.ac.uk/QuickGO/GTerm?id=GO:0006355) | Biological Process |
| Q7Z3A2 | Putative uncharacterized protein DKFZp686G1675 Fragment OS Homo sapiens GN DKFZp686G1675 PE 2 SV 1 | 254,41 | G2 | G2 | [Oxidoreductase activity, acting on the aldehyde or oxo group of donors, NAD or NADP as acceptor](http://www.ebi.ac.uk/QuickGO/GTerm?id=GO:0016620) | Molecular function |
| Q8IWY7 | Tau tubulin kinase OS Homo sapiens GN TTBK2 PE 1 SV 1 | 205,21 |  | 4,18 | [Regulation of cell shape](http://www.ebi.ac.uk/QuickGO/GTerm?id=GO:0008360) | Biological Process |
| Q8N532 | TUBA1C protein OS Homo sapiens GN TUBA1C PE 2 SV 1 | 29595,91 | G2 | 2,41 | [Microtubule-based process](http://www.ebi.ac.uk/QuickGO/GTerm?id=GO:0007017) | Biological Process |
| Q96IF9 | VCP protein Fragment OS Homo sapiens GN VCP PE 2 SV 2 | 215,19 | G2 | G2 | [ATP binding / hydrolase activity](http://www.ebi.ac.uk/QuickGO/GTerm?id=GO:0005524) | Molecular function |
| RAN | GTP binding nuclear protein Ran OS Homo sapiens GN RAN PE 1 SV 3 | 2156,6 | G2 | 1,14 | [Protein import into nucleus / small GTPase mediated signal transduction](http://www.ebi.ac.uk/QuickGO/GTerm?id=GO:0006606) | Biological Process |
| ROA2 | Heterogeneous nuclear ribonucleoproteins A2 B1 OS Homo sapiens GN HNRNPA2B1 PE 1 SV 2 | 836,38 |  | 1,51 | mRNA processing, mRNA splicing/ mRNA transport | Biological Process |
| RS3 | 40S ribosomal protein S3 OS Homo sapiens GN RPS3 PE 1 SV 2 | 1003,14 | G2 | G2 | Apoptosis, Cell cycle, Cell division, DNA damage, DNA repair, Mitosis, Transcription, Transcription regulation, Translation regulation | Biological Process |
| RS7 | 40S ribosomal protein S7 OS Homo sapiens GN RPS7 PE 1 SV 1 | 4372,39 |  | 1,36 | [Cellular protein metabolic process / gene expression / nuclear-transcribed mRNA catabolic process, nonsense-mediated decay / ribosomal small subunit biogenesis / viral life cycle](http://www.ebi.ac.uk/QuickGO/GTerm?id=GO:0044267) | Biological Process |
| SERPH | Serpin H1 OS Homo sapiens GN SERPINH1 PE 1 SV 2 | 188,62 | G2 | G2 | [Stress response](http://www.uniprot.org/keywords/KW-0346) | Biological Process |
| STML2 | Stomatin like protein 2 mitochondrial OS Homo sapiens GN STOML2 PE 1 SV 1 | 358,26 | G2 | 1,43 | [CD4-positive, alpha-beta T cell activation / cellular calcium ion homeostasis / interleukin-2 production / mitochondrial ATP synthesis coupled proton transport / positive regulation of cardiolipin metabolic process / stress-induced mitochondrial fusion / T cell receptor signaling pathway](http://www.ebi.ac.uk/QuickGO/GTerm?id=GO:0035710) | Biological Process |
| TALDO | Transaldolase OS Homo sapiens GN TALDO1 PE 1 SV 2 | 317,25 | G2 | G2 | [Pentose shunt](http://www.uniprot.org/keywords/KW-0570) | Biological Process |
| TBA3C | Tubulin alpha 3C D chain OS Homo sapiens GN TUBA3C PE 1 SV 3 | 13239,15 | G2 | G2 | [de novo' posttranslational protein folding / cellular protein metabolic process / microtubule-based process / protein folding](http://www.ebi.ac.uk/QuickGO/GTerm?id=GO:0051084) | Biological Process |
| TBA4B | Putative tubulin like protein alpha 4B OS Homo sapiens GN TUBA4B PE 5 SV 2 | 1400,42 | G2 | G2 | [Microtubule-based process](http://www.ebi.ac.uk/QuickGO/GTerm?id=GO:0007017) | Biological Process |
| TBA8 | Tubulin alpha 8 chain OS Homo sapiens GN TUBA8 PE 1 SV 1 | 4185,04 | G2 | 6,62 | [Microtubule-based process](http://www.ebi.ac.uk/QuickGO/GTerm?id=GO:0007017) | Biological Process |
| TBB8 | Tubulin beta 8 chain OS Homo sapiens GN TUBB8 PE 1 SV 2 | 2246,36 | G2 | G2 | [Microtubule-based process](http://www.ebi.ac.uk/QuickGO/GTerm?id=GO:0007017) | Biological Process |
| TKT | Transketolase OS Homo sapiens GN TKT PE 1 SV 3 | 5900,67 | G2 | G2 | Carbohydrate metabolic process / energy reserve metabolic process / glyceraldehyde-3-phosphate biosynthetic process / pentose-phosphate shunt / pentose-phosphate shunt, non-oxidative branch / regulation of growth / small molecule metabolic process / xylulose biosynthetic process | Biological Process |
| U3KQK0 | Histone H2B OS Homo sapiens GN HIST1H2BN PE 1 SV 1 | 8878,4 | G2 | G2 | [Nucleosome assembly](http://www.ebi.ac.uk/QuickGO/GTerm?id=GO:0006334) | Biological Process |
| UBE2N | Ubiquitin conjugating enzyme E2 N OS Homo sapiens GN UBE2N PE 1 SV 1 | 774,63 | G2 | G2 | DNA damage, DNA repair, Ubl conjugation pathway | Biological Process |
| UBQL1 | Ubiquilin 1 OS Homo sapiens GN UBQLN1 PE 1 SV 2 | 160,88 | G2 | G2 | [Autophagy](http://www.uniprot.org/keywords/KW-0072) | Biological Process |
| V9HWK1 | Triosephosphate isomerase OS Homo sapiens GN HEL S 49 PE 2 SV 1 | 3128,38 |  | 1,08 | [Gluconeogenesis](http://www.uniprot.org/keywords/KW-0312) | Biological Process |
| VIME | Vimentin OS Homo sapiens GN VIM PE 1 SV 4 | 2186,5 |  | 1,35 | [Host-virus interaction](http://www.uniprot.org/keywords/KW-0945) | Biological Process |
