# Supplementary material for: Poly-lactic acid nanoparticles (PLA-NP) promote physiological modifications in lung epithelial cells and are internalized by clathrin-coated pits and lipid rafts
Source: J Nanobiotechnology. 2017 Jan 31;15:11. doi: 10.1186/s12951-016-0238-1 (PMC5282631; doi:10.1186/s12951-016-0238-1)
Supplement: Supplementary file 2 — Additional file 2: Table S2. Downregulated polypeptides identified by mass spectrometry from A549 proteome in response to PLA-NP with their respective description. [file 12951_2016_238_MOESM2_ESM.docx]

| **Downregulated polypeptides identified by mass spectrometry from A549 proteome in response to PLA-NP** | | | | | | |
| --- | --- | --- | --- | --- | --- | --- |
| **Accession** | **Description** | **Id Score** | **Unique** | **G2/G1** | **GO Function** | **GO Category** |
| 1433B | 14 3 3 protein beta alpha OS Homo sapiens GN YWHAB PE 1 SV 3 | 3293,23 | G1 | 0,73 | [Host-virus interaction](http://www.uniprot.org/keywords/KW-0945) | Biological Process |
| 6PGD | 6 phosphogluconate dehydrogenase decarboxylating OS Homo sapiens GN PGD PE 1 SV 3 | 1971,61 | G1 | 0,43 | Gluconate utilization, Pentose shunt | Biological Process |
| A0A024R5Z9 | Pyruvate kinase OS Homo sapiens GN PKM2 PE 3 SV 1 | 3188,68 | G1 | G1 | [Glycolysis](http://www.uniprot.org/keywords/KW-0324) | Biological Process |
| A0A024R7F1 | Protein kinase C substrate 80K H isoform CRA a OS Homo sapiens GN PRKCSH PE 4 SV 1 | 135,69 | G1 | G1 | [N-glycan processing](http://www.ebi.ac.uk/QuickGO/GTerm?id=GO:0006491) | Biological Process |
| A0A024R895 | SET translocation Myeloid leukemia associated isoform CRA b OS Homo sapiens GN SET PE 3 SV 1 | 1382,17 | G1 | 0,15 | [Nucleosome assembly](http://www.ebi.ac.uk/QuickGO/GTerm?id=GO:0006334) | Biological Process |
| A0A024RAV4 | Cold shock domain protein A isoform CRA b OS Homo sapiens GN CSDA PE 4 SV 1 | 2567,37 | G1 | G1 | [Regulation of transcription (DNA-templated)](http://www.ebi.ac.uk/QuickGO/GTerm?id=GO:0006355) | Biological Process |
| A0A075B759 | Peptidyl prolyl cis trans isomerase OS Homo sapiens GN PPIAL4F PE 3 SV 1 | 2652 | G1 | G1 | None | None |
| A0A087WUM2 | L lactate dehydrogenase OS Homo sapiens GN LDHAL6A PE 3 SV 1 | 795,19 | G1 | 0,09 | [Carbohydrate metabolic process (carboxylic acid metabolic process)](http://www.ebi.ac.uk/QuickGO/GTerm?id=GO:0005975) | Biological Process |
| A0A0A0MTS2 | Glucose 6 phosphate isomerase Fragment OS Homo sapiens GN GPI PE 4 SV 1 | 894,84 | G1 | G1 | [Gluconeogenesis](http://www.uniprot.org/keywords/KW-0312) | Biological Process |
| A0AUL6 | ACTB protein Fragment OS Homo sapiens GN ACTB PE 2 SV 1 | 6644,29 | G1 | 0,32 | None | None |
| A4D1P0 | Uncharacterized protein OS Homo sapiens GN LOC402299 PE 4 SV 1 | 3471,17 | G1 | 0,42 | [Oxidoreductase activity](http://www.ebi.ac.uk/QuickGO/GTerm?id=GO:0016491) | Molecular function |
| A6NLJ7 | Ubiquitin carboxyl terminal hydrolase OS Homo sapiens GN UCHL1 PE 2 SV 1 | 622,07 | G1 | G1 | [Ubl conjugation pathway](http://www.uniprot.org/keywords/KW-0833) | Biological Process |
| A8MUF7 | Hemoglobin subunit epsilon Fragment OS Homo sapiens GN HBE1 PE 1 SV 1 | 14077,35 | G1 | G1 | [Oxygen transport](http://www.uniprot.org/keywords/KW-0561) | Biological Process |
| ACTB | Actin cytoplasmic 1 OS Homo sapiens GN ACTB PE 1 SV 1 | 91933,19 | G1 | 0,7 | [de novo' posttranslational protein folding / adherens junction organization / ATP-dependent chromatin remodeling / axon guidance / blood coagulation / cell-cell junction organization / vascular endothelial growth factor receptor signaling pathway ... entre outros](http://www.ebi.ac.uk/QuickGO/GTerm?id=GO:0051084) | Biological Process |
| ACTN4 | Alpha actinin 4 OS Homo sapiens GN ACTN4 PE 1 SV 2 | 4953,46 |  | 0,82 | Protein transport | Biological Process |
| AL1A1 | Retinal dehydrogenase 1 OS Homo sapiens GN ALDH1A1 PE 1 SV 2 | 10628,41 |  | 0,73 | Cellular aldehyde metabolic process / ethanol oxidation / positive regulation of GTPase activity / retinol metabolic process / small molecule metabolic process / xenobiotic metabolic process | Biological Process |
| ANXA1 | Annexin A1 OS Homo sapiens GN ANXA1 PE 1 SV 2 | 23748,67 |  | 0,56 | Immunity, Inflammatory response | Biological Process |
| ANXA5 | Annexin A5 OS Homo sapiens GN ANXA5 PE 1 SV 2 | 13664,37 |  | 0,21 | Blood coagulation, Hemostasis | Biological Process |
| B2M1S8 | Delta globin B2 variant Fragment OS Homo sapiens GN HBD PE 3 SV 1 | 11571,07 | G1 | G1 | [Oxygen transport](http://www.uniprot.org/keywords/KW-0561) | Biological Process |
| B2R6Y1 | Histone H3 OS Homo sapiens PE 2 SV 1 | 1182,7 | G1 | G1 | [DNA binding](http://www.ebi.ac.uk/QuickGO/GTerm?id=GO:0003677) | Molecular function |
| B3KQT2 | Protein disulfide isomerase OS Homo sapiens PE 2 SV 1 | 286,1 | G1 | G1 | [Cell redox homeostasis](http://www.ebi.ac.uk/QuickGO/GTerm?id=GO:0045454) | Biological Process |
| B4DM82 | Peptidyl prolyl cis trans isomerase OS Homo sapiens PE 2 SV 1 | 7676,22 | G1 | 0,86 | [Protein folding](http://www.ebi.ac.uk/QuickGO/GTerm?id=GO:0006457) | Biological Process |
| B4DMJ7 | HCG2015269 isoform CRA c OS Homo sapiens GN hCG 2015269 PE 2 SV 1 | 881,88 | G1 | G1 | [Glycolytic process](http://www.ebi.ac.uk/QuickGO/GTerm?id=GO:0006096) | Biological Process |
| B4DRV9 | Glyceraldehyde 3 phosphate dehydrogenase OS Homo sapiens PE 2 SV 1 | 11573,8 | G1 | 0,15 | Oxidoreductase | Molecular function |
| B4DUI5 | Triosephosphate isomerase OS Homo sapiens PE 2 SV 1 | 5740,28 | G1 | G1 | [Gluconeogenesis](http://www.uniprot.org/keywords/KW-0312) | Biological Process |
| B7Z1Y2 | Fructose bisphosphate aldolase OS Homo sapiens PE 2 SV 1 | 1233,52 | G1 | G1 | Glycolysis | Biological Process |
| B7Z596 | Tropomyosin alpha 1 chain OS Homo sapiens GN TPM1 PE 1 SV 1 | 3322,34 | G1 | G1 | Muscle contraction / muscle filament sliding | None |
| B7ZW15 | Uncharacterized protein OS Homo sapiens PE 2 SV 1 | 912,47 | G1 | G1 | Glycolytic process | Biological Process |
| B8ZZA1 | Thymosin alpha 1 OS Homo sapiens GN PTMA PE 1 SV 1 | 968,82 | G1 | G1 | None | None |
| B8ZZQ6 | Uncharacterized protein OS Homo sapiens GN PTMA PE 1 SV 1 | 1044,46 | G1 | G1 | None | None |
| B8ZZW7 | Thymosin alpha 1 OS Homo sapiens GN PTMA PE 1 SV 1 | 967,91 | G1 | G1 | None | None |
| BAF | Barrier to autointegration factor OS Homo sapiens GN BANF1 PE 1 SV 1 | 3405,89 | G1 | 0,63 | Host-virus interaction | Biological Process |
| BTF3 | Transcription factor BTF3 OS Homo sapiens GN BTF3 PE 1 SV 1 | 2683,77 | G1 | 0,76 | Protein transport, Transcription, Transcription regulation, Transport | Biological Process |
| C9J4W5 | Eukaryotic translation initiation factor 5A 2 Fragment OS Homo sapiens GN EIF5A2 PE 4 SV 1 | 561,21 | G1 | G1 | [Protein biosynthesis](http://www.uniprot.org/keywords/KW-0648) | Biological Process |
| CALL3 | Calmodulin like protein 3 OS Homo sapiens GN CALML3 PE 1 SV 2 | 2686,24 | G1 | G1 | Calcium ion binding | Molecular function |
| CALM | Calmodulin OS Homo sapiens GN CALM1 PE 1 SV 2 | 36602,48 |  | 0,17 | Calcium ion binding | Molecular function |
| CALR | Calreticulin OS Homo sapiens GN CALR PE 1 SV 1 | 11346,83 | G1 | G1 | Antigen processing and presentation of exogenous peptide antigen via MHC class I / antigen processing and presentation of exogenous peptide antigen via MHC class I, TAP-dependent / antigen processing and presentation of peptide antigen via MHC class I / cell cycle arrest / and others | Biological Process |
| CALU | Calumenin OS Homo sapiens GN CALU PE 1 SV 2 | 847,16 | G1 | 0,72 | [Blood coagulation / peripheral nervous system axon regeneration / platelet activation / platelet degranulation / response to organic cyclic compound](http://www.ebi.ac.uk/QuickGO/GTerm?id=GO:0007596) | Biological Process |
| CH10 | 10 kDa heat shock protein mitochondrial OS Homo sapiens GN HSPE1 PE 1 SV 2 | 68713,13 |  | 0,15 | Stress response | Biological Process |
| COF1 | Cofilin 1 OS Homo sapiens GN CFL1 PE 1 SV 3 | 3880,74 |  | 0,63 | [Actin filament depolymerization](http://www.ebi.ac.uk/QuickGO/GTerm?id=GO:0030042) | Biological Process |
| D6RHD5 | Serum albumin OS Homo sapiens GN ALB PE 1 SV 1 | 176,38 | G1 | G1 | Transport | Biological Process |
| D9YZV5 | Tropomyosin 1 Alpha isoform 4 OS Homo sapiens GN TPM1 PE 3 SV 1 | 4348,24 | G1 | 0,58 | Muscle contraction / muscle filament sliding | Biological Process |
| E7ETZ0 | Calmodulin OS Homo sapiens GN CALM1 PE 1 SV 1 | 36602,48 | G1 | 0,17 | Calcium ion binding | Molecular function |
| E9PK01 | Elongation factor 1 delta Fragment OS Homo sapiens GN EEF1D PE 1 SV 1 | 8231,05 | G1 | 0,68 | Protein biosynthesis | Biological Process |
| E9PK25 | Cofilin 1 OS Homo sapiens GN CFL1 PE 1 SV 1 | 12077,89 | G1 | 0,65 | [Actin filament depolymerization](http://www.ebi.ac.uk/QuickGO/GTerm?id=GO:0030042) | Biological Process |
| EF2 | Elongation factor 2 OS Homo sapiens GN EEF2 PE 1 SV 4 | 515,7 | G1 | 0,61 | Protein biosynthesis | Biological Process |
| EFTU | Elongation factor Tu mitochondrial OS Homo sapiens GN TUFM PE 1 SV 2 | 222,02 |  | 0,77 | Protein biosynthesis | Biological Process |
| F5H5G7 | L lactate dehydrogenase OS Homo sapiens GN LDHC PE 3 SV 1 | 1449,94 | G1 | 0,2 | [Carbohydrate metabolic process (carboxylic acid metabolic process)](http://www.ebi.ac.uk/QuickGO/GTerm?id=GO:0005975) | Biological Process |
| F6KPG5 | Albumin Fragment OS Homo sapiens PE 2 SV 1 | 2617,09 | G1 | G1 | Transport | Biological Process |
| F6S2S5 | LIM and SH3 domain protein 1 OS Homo sapiens GN LASP1 PE 1 SV 1 | 742,47 | G1 | G1 | Zinc ion binding | Molecular function |
| F6THM6 | Alpha actinin 2 OS Homo sapiens GN ACTN2 PE 1 SV 1 | 521,13 | G1 | G1 | Calcium ion binding | Molecular function |
| F8VXI6 | Nucleosome assembly protein 1 like 1 Fragment OS Homo sapiens GN NAP1L1 PE 1 SV 1 | 4109,36 | G1 | G1 | Nucleosome assembly | Biological Process |
| F8VY35 | Nucleosome assembly protein 1 like 1 Fragment OS Homo sapiens GN NAP1L1 PE 1 SV 1 | 4104,84 |  | 0,35 | Nucleosome assembly | Biological Process |
| F8W0F6 | Tubulin alpha 1A chain OS Homo sapiens GN TUBA1A PE 4 SV 1 | 11427,42 | G1 | 0,35 | Microtubule-based process | Biological Process |
| F8W1U3 | Keratin type II cytoskeletal 8 Fragment OS Homo sapiens GN KRT8 PE 1 SV 1 | 4115,64 | G1 | G1 | Structural molecule activity | Molecular function |
| FETUA | Alpha 2 HS glycoprotein OS Homo sapiens GN AHSG PE 1 SV 1 | 5404,44 |  | 0,88 | Mineral balance | Biological Process |
| G8JLA2 | Myosin light polypeptide 6 OS Homo sapiens GN MYL6 PE 1 SV 1 | 9475,93 |  | 0,28 | Calcium ion binding | Molecular function |
| H0Y2Y8 | Zyxin Fragment OS Homo sapiens GN ZYX PE 1 SV 1 | 155,73 | G1 | G1 | Zinc ion binding | Molecular function |
| H0Y8K3 | Protein CDV3 homolog Fragment OS Homo sapiens GN CDV3 PE 1 SV 1 | 953,16 | G1 | G1 | None | None |
| H0YIV4 | Nucleosome assembly protein 1 like 1 Fragment OS Homo sapiens GN NAP1L1 PE 1 SV 1 | 987,27 | G1 | G1 | Nucleosome assembly | Biological Process |
| H0YLU7 | Electron transfer flavoprotein subunit alpha mitochondrial Fragment OS Homo sapiens GN ETFA PE 1 | 168,95 | G1 | G1 | None | None |
| H2AX | Histone H2AX OS Homo sapiens GN H2AFX PE 1 SV 2 | 46989,26 | G1 | 0,28 | Cell cycle, DNA damage, DNA recombination, DNA repair, Host-virus interaction, Meiosis | Biological Process |
| H7BYY1 | Tropomyosin 1 Alpha isoform CRA m OS Homo sapiens GN TPM1 PE 1 SV 1 | 3449,15 | G1 | 0,06 | None | None |
| H90B2 | Putative heat shock protein HSP 90 beta 2 OS Homo sapiens GN HSP90AB2P PE 1 SV 2 | 1886,87 |  | 0,16 | Stress response | Biological Process |
| HBA_BOVIN | HEMOGLOBIN ALPHA CHAIN | 3265,29 |  | 0,94 | Oxygen transport | Biological Process |
| HBG1 | Hemoglobin subunit gamma 1 OS Homo sapiens GN HBG1 PE 1 SV 2 | 14069,3 | G1 | G1 | Oxygen transport | Biological Process |
| HN1 | Hematological and neurological expressed 1 protein OS Homo sapiens GN HN1 PE 1 SV 3 | 4602,08 | G1 | 0,54 | None | None |
| HSP72 | Heat shock related 70 kDa protein 2 OS Homo sapiens GN HSPA2 PE 1 SV 1 | 8009,42 |  | 0,39 | Stress response | Biological Process |
| HSP76 | Heat shock 70 kDa protein 6 OS Homo sapiens GN HSPA6 PE 1 SV 2 | 7695,44 | G1 | 0,29 | Stress response | Biological Process |
| I3L397 | Eukaryotic translation initiation factor 5A 1 Fragment OS Homo sapiens GN EIF5A PE 1 SV 3 | 504,16 |  | 0,42 | Protein biosynthesis | Biological Process |
| I3L3D5 | Profilin 1 Fragment OS Homo sapiens GN PFN1 PE 1 SV 1 | 6221,94 | G1 | 0,47 | [Actin cytoskeleton organization](http://www.ebi.ac.uk/QuickGO/GTerm?id=GO:0030036) | Biological Process |
| I3L4N8 | Actin cytoplasmic 2 Fragment OS Homo sapiens GN ACTG1 PE 1 SV 3 | 67402,91 | G1 | 0,66 | None | None |
| J3KT65 | Actin cytoplasmic 2 N terminally processed OS Homo sapiens GN ACTG1 PE 1 SV 1 | 29970,98 | G1 | G1 | ATP binding | Molecular function |
| J3QL05 | Serine arginine rich splicing factor 2 Fragment OS Homo sapiens GN SRSF2 PE 1 SV 1 | 8208,92 | G1 | G1 | Nucleic acid binding / nucleotide binding | Molecular function |
| J3QRK5 | Protein UBBP4 OS Homo sapiens GN UBBP4 PE 1 SV 1 | 5462,49 | G1 | 0,15 | None | None |
| K2C5 | Keratin type II cytoskeletal 5 OS Homo sapiens GN KRT5 PE 1 SV 3 | 249,11 |  | 0,13 | [Cell junction assembly / epidermis development / hemidesmosome assembly](http://www.ebi.ac.uk/QuickGO/GTerm?id=GO:0034329) | Biological Process |
| K4EQ44 | Beta actin Fragment OS Homo sapiens PE 4 SV 1 | 4955,02 | G1 | G1 | None | None |
| K7EJH8 | Alpha actinin 4 Fragment OS Homo sapiens GN ACTN4 PE 1 SV 1 | 122,76 | G1 | G1 | [Bicellular tight junction assembly / protein localization to bicellular tight junction](http://www.ebi.ac.uk/QuickGO/GTerm?id=GO:0070830) | Biological Process |
| K7EP68 | Tropomyosin alpha 4 chain Fragment OS Homo sapiens GN TPM4 PE 1 SV 1 | 1356,54 | G1 | G1 | Muscle contraction / muscle filament sliding | None |
| K7EQJ5 | 40S ribosomal protein S15 OS Homo sapiens GN RPS15 PE 1 SV 2 | 524,36 | G1 | G1 | [Ribosome biogenesis / translation](http://www.ebi.ac.uk/QuickGO/GTerm?id=GO:0000028) | Biological Process |
| K7ESM5 | Tubulin beta 6 chain Fragment OS Homo sapiens GN TUBB6 PE 1 SV 1 | 1432,39 | G1 | G1 | Microtubule-based process | Biological Process |
| KPYM | Pyruvate kinase PKM OS Homo sapiens GN PKM PE 1 SV 4 | 33613,23 |  | 0,84 | Glycolysis | Biological Process |
| LASP1 | LIM and SH3 domain protein 1 OS Homo sapiens GN LASP1 PE 1 SV 2 | 3769,18 | G1 | 0,16 | Ion transport, transport | Biological Process |
| LDH6A | L lactate dehydrogenase A like 6A OS Homo sapiens GN LDHAL6A PE 2 SV 1 | 2273,51 | G1 | 0,56 | [Carbohydrate metabolic process (carboxylic acid metabolic process)](http://www.ebi.ac.uk/QuickGO/GTerm?id=GO:0005975) | Biological Process |
| LEG1 | Galectin 1 OS Homo sapiens GN LGALS1 PE 1 SV 2 | 946,39 |  | 0,73 | Apoptosis | Biological Process |
| MYL6B | Myosin light chain 6B OS Homo sapiens GN MYL6B PE 1 SV 1 | 3406,11 | G1 | G1 | [Muscle contraction / muscle filament sliding](http://www.ebi.ac.uk/QuickGO/GTerm?id=GO:0006936) | Biological Process |
| NDK8 | Putative nucleoside diphosphate kinase OS Homo sapiens GN NME2P1 PE 5 SV 1 | 407,45 | G1 | G1 | Nucleotide metabolism | Biological Process |
| NDKA | Nucleoside diphosphate kinase A OS Homo sapiens GN NME1 PE 1 SV 1 | 1893,1 | G1 | G1 | Differentiation, Endocytosis, Neurogenesis, Nucleotide metabolism | Biological Process |
| NP1L4 | Nucleosome assembly protein 1 like 4 OS Homo sapiens GN NAP1L4 PE 1 SV 1 | 402,32 | G1 | G1 | Nucleosome assembly | Biological Process |
| NPM | Nucleophosmin OS Homo sapiens GN NPM1 PE 1 SV 2 | 18698,73 |  | 0,34 | Host-virus interaction | Biological Process |
| NSF1C | NSFL1 cofactor p47 OS Homo sapiens GN NSFL1C PE 1 SV 2 | 2162,67 | G1 | G1 | [Autophagosome assembly / Golgi organization / membrane fusion / nuclear envelope reassembly / proteasome-mediated ubiquitin-dependent protein catabolic process / regulation of catalytic activity](http://www.ebi.ac.uk/QuickGO/GTerm?id=GO:0000045) | Biological Process |
| NSF1C | NSFL1 cofactor p47 OS Homo sapiens GN NSFL1C PE 1 SV 2 | 605,38 |  | 0,66 | [Autophagosome assembly / Golgi organization / membrane fusion / nuclear envelope reassembly / proteasome-mediated ubiquitin-dependent protein catabolic process / regulation of catalytic activity](http://www.ebi.ac.uk/QuickGO/GTerm?id=GO:0000045) | Biological Process |
| PARK7 | Protein DJ 1 OS Homo sapiens GN PARK7 PE 1 SV 2 | 11211,07 |  | 0,31 | Autophagy, Fertilization, Inflammatory response, Stress response | Biological Process |
| PDCD5 | Programmed cell death protein 5 OS Homo sapiens GN PDCD5 PE 1 SV 3 | 2002,82 | G1 | 0,17 | Apoptosis | Biological Process |
| PDLI1 | PDZ and LIM domain protein 1 OS Homo sapiens GN PDLIM1 PE 1 SV 4 | 565,85 |  | 0,28 | [Regulation of transcription, DNA-templated / response to hypoxia / response to oxidative stress](http://www.ebi.ac.uk/QuickGO/GTerm?id=GO:0006355) | Biological Process |
| PGAM1 | Phosphoglycerate mutase 1 OS Homo sapiens GN PGAM1 PE 1 SV 2 | 1454,74 | G1 | 0,7 | Glycolysis | Biological Process |
| POTEI | POTE ankyrin domain family member I OS Homo sapiens GN POTEI PE 3 SV 1 | 9237,79 |  | 0,2 | Retina homeostasis | Biological Process |
| PRDX2 | Peroxiredoxin 2 OS Homo sapiens GN PRDX2 PE 1 SV 5 | 612,31 | G1 | G1 | [Activation of MAPK activity / cellular response to oxidative stress / gene expression / homeostasis of number of cells / negative regulation of apoptotic process / negative regulation of NF-kappaB transcription factor activity / entre outros...](http://www.ebi.ac.uk/QuickGO/GTerm?id=GO:0000187) | Biological Process |
| PTMS | Parathymosin OS Homo sapiens GN PTMS PE 1 SV 2 | 12465,13 | G1 | G1 | Immunity | Biological Process |
| Q14222 | EEF1A protein Fragment OS Homo sapiens GN EEF1A PE 1 SV 1 | 4148,9 | G1 | G1 | Protein biosynthesis | Biological Process |
| Q2TUW9 | Lactoferrin OS Homo sapiens PE 2 SV 1 | 542,49 | G1 | G1 | [Antibacterial humoral response / antifungal humoral response / regulation of cytokine production](http://www.ebi.ac.uk/QuickGO/GTerm?id=GO:0019731) | Biological Process |
| Q2XPP3 | Type II 3a hydroxysteroid dehydrogenase variant OS Homo sapiens PE 2 SV 1 | 9285,02 | G1 | 0,91 | [Oxidoreductase activity](http://www.ebi.ac.uk/QuickGO/GTerm?id=GO:0016491) | Molecular function |
| Q53GE9 | Elongation factor 1 alpha Fragment OS Homo sapiens PE 2 SV 1 | 6234,11 | G1 | G1 | Protein biosynthesis | Biological Process |
| Q562M5 | Actin like protein Fragment OS Homo sapiens GN ACT PE 3 SV 1 | 860,88 | G1 | G1 | None | None |
| Q562V5 | Actin like protein Fragment OS Homo sapiens GN ACT PE 3 SV 1 | 16762,67 | G1 | 0,14 | None | None |
| Q562X0 | Actin like protein Fragment OS Homo sapiens GN ACT PE 3 SV 1 | 4405,57 | G1 | G1 | None | None |
| Q59EJ3 | Heat shock 70kDa protein 1A variant Fragment OS Homo sapiens PE 2 SV 1 | 12746,03 | G1 | 0,38 | Stress response | Biological Process |
| Q59EM9 | Ubiquitin C variant Fragment OS Homo sapiens PE 2 SV 1 | 2618,65 |  | 0,36 | None | None |
| Q59FD9 | Actinin alpha 2 variant Fragment OS Homo sapiens PE 2 SV 1 | 535,55 | G1 | 0,14 | Calcium ion binding | Molecular function |
| Q59H57 | Fusion Involved in t 12 16 in malignant liposarcoma isoform a variant Fragment OS Homo sapiens | 2695,79 | G1 | G1 | Nucleic acid binding / nucleotide binding / zinc ion binding | Molecular function |
| Q670S4 | Hemoglobin Lepore Baltimore Fragment OS Homo sapiens PE 3 SV 1 | 14055,19 | G1 | G1 | Oxygen transport | Biological Process |
| Q6E433 | Activated RNA polymerase II transcription cofactor 4 Fragment OS Homo sapiens PE 2 SV 1 | 1064,75 | G1 | G1 | Regulation of transcription (DNA - templated) | Biological Process |
| Q6IPT9 | Elongation factor 1 alpha OS Homo sapiens GN EEF1A1 PE 2 SV 1 | 6520,66 | G1 | 0,33 | Protein biosynthesis | Biological Process |
| Q6QMJ5 | Tubulin alpha 1 Fragment OS Homo sapiens GN TUBA1 PE 2 SV 1 | 640,39 | G1 | G1 | Microtubule-based process | Biological Process |
| Q71V99 | Peptidyl prolyl cis trans isomerase OS Homo sapiens PE 2 SV 1 | 4729,31 | G1 | G1 | Protein folding | Biological Process |
| Q7Z612 | Acidic ribosomal phosphoprotein P1 OS Homo sapiens PE 2 SV 1 | 18173,52 |  | 0,36 | Translation | Biological Process |
| Q7Z7J6 | Actin alpha 1 skeletal muscle protein OS Homo sapiens GN ACTA1 PE 2 SV 1 | 20291,86 | G1 | G1 | ATP binding | Molecular function |
| Q96RS2 | 40S ribosomal protein SA OS Homo sapiens GN RPSA PE 2 SV 1 | 980,83 | G1 | G1 | Cell adhesion / ribosome biogenesis / translation | Biological Process |
| Q9NZE6 | BM 010 OS Homo sapiens GN EIF4A2 PE 2 SV 1 | 99,4 | G1 | G1 | Protein biosynthesis | Biological Process |
| RCN1 | Reticulocalbin 1 OS Homo sapiens GN RCN1 PE 1 SV 1 | 2025,89 | G1 | G1 | Camera-type eye development / in utero embryonic development | Biological Process |
| RLA1 | 60S acidic ribosomal protein P1 OS Homo sapiens GN RPLP1 PE 1 SV 1 | 18173,52 | G1 | 0,32 | Cellular protein metabolic process / gene expression / nuclear-transcribed mRNA catabolic process, nonsense-mediated decay / SRP-dependent cotranslational protein targeting to membrane / translation / viral life cycle / entre outros... | Biological Process |
| RLA2 | 60S acidic ribosomal protein P2 OS Homo sapiens GN RPLP2 PE 1 SV 1 | 70134,35 |  | 0,15 | Cellular protein metabolic process / gene expression / nuclear-transcribed mRNA catabolic process, nonsense-mediated decay / SRP-dependent cotranslational protein targeting to membrane / translation / viral life cycle / entre outros... | Biological Process |
| S10AB | Protein S100 A11 OS Homo sapiens GN S100A11 PE 1 SV 2 | 14318,45 |  | 0,2 | Negative regulation of cell proliferation / negative regulation of DNA replication / signal transduction | Biological Process |
| S4R3N1 | Protein HSPE1 MOB4 OS Homo sapiens GN HSPE1 MOB4 PE 3 SV 1 | 21665,1 |  | 0,14 | Protein folding | Biological Process |
| S4R3Z2 | Aldo keto reductase family 1 member C3 OS Homo sapiens GN AKR1C3 PE 1 SV 1 | 429,07 | G1 | G1 | Oxidoreductase activity | Molecular function |
| SODC | Superoxide dismutase Cu Zn OS Homo sapiens GN SOD1 PE 1 SV 2 | 732,5 |  | 0,31 | Activation of MAPK activity / anterograde axon cargo transport / blood coagulation / cell aging / cellular response to ATP / response to reactive oxygen species / entre outros .. | Biological Process |
| STMN1 | Stathmin OS Homo sapiens GN STMN1 PE 1 SV 3 | 5291,15 | G1 | G1 | Differentiation, Neurogenesis | Biological Process |
| TPM2 | Tropomyosin beta chain OS Homo sapiens GN TPM2 PE 1 SV 1 | 4301,02 | G1 | G1 | Muscle contraction / muscle filament sliding | Biological Process |
| TPM4 | Tropomyosin alpha 4 chain OS Homo sapiens GN TPM4 PE 1 SV 3 | 3106,27 | G1 | G1 | Muscle contraction / muscle filament sliding | Biological Process |
| U6A216 | Mutant hemoglobin alpha 1 globin chain Fragment OS Homo sapiens GN HBA1 PE 3 SV 1 | 1034,07 | G1 | G1 | Oxygen transport | Biological Process |
| UCHL1 | Ubiquitin carboxyl terminal hydrolase isozyme L1 OS Homo sapiens GN UCHL1 PE 1 SV 2 | 2133,45 | G1 | 0,68 | Ubl conjugation pathway | Biological Process |
| V9HVX6 | Epididymis luminal protein 9 OS Homo sapiens GN HEL 9 PE 2 SV 1 | 10628,41 | G1 | 0,76 | Oxidoreductase activity | Molecular function |
| V9HW22 | Epididymis luminal protein 33 OS Homo sapiens GN HEL S 72p PE 2 SV 1 | 7925,43 | G1 | G1 | Stress response | Biological Process |
|  |  |  |  |  |  |  |
